# Does Quality Affect Patients’ Choice of Doctor? Evidence from England

**DOI:** 10.1111/ecoj.12282

**Published:** 2016-02-23

**Authors:** Rita Santos, Hugh Gravelle, Carol Propper

**Affiliations:** ^1^ University of York; ^2^ University of BristolImperial College London and CEPR

## Abstract

Reforms giving users of public services choice of provider aim to improve quality. But such reforms will work only if quality affects choice of provider. We test this crucial prerequisite in the English health care market by examining the choice of 3.4 million individuals of family doctor. Family doctor practices provide primary care and control access to non‐emergency hospital care, the quality of their clinical care is measured and published and care is free. In this setting, clinical quality should affect choice. We find that a 1 standard deviation increase in clinical quality would increase practice size by around 17%.

Governments in the UK and internationally have increasingly turned to policies to create or enhance consumer choice for public services including education, employment services, health care, public housing and social care (Besley and Ghatak, 2003; Pawson *et al*., 2006; Frontier Economics, 2010; Musset, 2012; Vrangbaek *et al*., 2012). Giving consumers the power to choose their public service supplier, it is argued, will produce a better match of consumers and suppliers, and give suppliers an incentive to provide higher quality (Hoxby, 2003; Le Grand, 2003).

Choice is a popular reform model in health care, adopted by administrations of different political orientation, including the USA, the UK, Denmark, Italy (Lombardy), the Netherlands, Germany and Sweden (Thompson and Dixon, 2006). A necessary condition for greater patient choice to improve quality is that a provider will face higher demand if they improve their quality. This is the question we address here. We do so by examining the choice of family doctor in the English National Health Service (NHS). This is an important setting. First, all individuals in the UK are entitled to choose an NHS family doctor practice and need to do so, as family doctors provide almost all primary care and are also the gatekeepers for any specialist or non‐emergency hospital care the individual may need. Primary care is where most people will have regular interactions with the health care system. Thus, the decision is salient for a large number of people. Second, good quality treatment by primary care physicians can prevent declines in health that result in the need for more expensive secondary care and family doctors guide patients to the most appropriate provider when secondary care is required. Quality of primary care therefore has important implications for both patient health and welfare and the public finances. Third, primary care is free at point of use, so whether individuals take account of quality when choosing a family doctor is more likely to be revealed in this setting. Fourth, because of the introduction of a pay for performance scheme in 2004 (Roland, 2004), there is uniquely good, and publically available, data on clinical quality for all English general practices. Finally, many other health care systems have similar settings – gatekeeping primary care providers and low direct monetary costs of using primary care (Saltman *et al*., 2006) – so that evidence from the UK has wider relevance.

We use data on the choices made by nearly 3.4 million adults aged 25 and over from amongst nearly 1,000 family doctor practices to examine the determinants of choice of practice and, in particular, to test whether quality affects choice. Our data contain a rich set of measures of practice quality, as well as information on the distances from patients to potential practices, and characteristics of the practice which have been shown to influence choice of patients, including the age and gender of the doctors in the practice, their country of qualification and the type of contract the practice has with the NHS.^1^

We find that individuals are more likely to choose practices which are of higher quality as measured by publicly available data on practice performance. The positive effect of clinical quality on choice is robust across age and gender groups, to the socio‐economic characteristics of the small areas in which the individuals reside, to allowing for unobserved heterogeneity in preferences and to the potential endogeneity of the clinical quality measure. We also find that distance is important – as expected given that health care has a strong local dimension – so valuation of practices decreases with distance from the individual's home. People are also more likely to choose practices which have a higher proportion of general practitioners (GPs) qualified in Europe, a higher proportion of female GPs and a lower average GP age.

The responsiveness of choice to practice quality is economically meaningful as well as statistically significant. The relevant effect for assessing the potential incentive for practices to improve quality is the increase in the number of individuals who wish to join a practice when its quality increases. Around 25,000 adults aged 25 and over live within 2 km of the average practice, so even small changes in the probability that an individual will choose a practice can lead to a sizeable increase in the practice list. Using our most conservative estimates, an increase of 1 standard deviation (SD) in clinical quality will increase the number of individuals over the age of 25 choosing a practice by just over 900, or around 17% of the mean number of such patients in a practice.

Our results contribute to the literature on choice in health care.^2^ Most studies that have examined the effect of quality have been undertaken in the context of choice of hospital rather than of family doctor. US‐based studies of patient choice of hospital (Luft *et al*., 1990; Burns and Wholey, 1992; Tay, 2003; Cutler *et al*., 2004; Howard, 2005; Ho, 2006; Pope, 2009) find that higher hospital quality increases demand. Similar findings have been reported for the Netherlands (Varkevisser *et al*., 2012), Italy (Moscone *et al*., 2012) and England (Beckert *et al*., 2012; Gaynor *et al*., 2012; Sivey, 2012). In the US, the introduction of report cards for health insurance plans and hospitals have led patients to choose better quality plans and providers (Scanlon *et al*., 2002; Wedig and Tai‐Seale, 2002; Kolstad and Chernew, 2009).

There are few studies of the effects of quality on patient choice of family doctors, perhaps because of the previous lack of good data on quality. Research has focused on other attributes of care or proxies for quality. Studies have shown the importance of distance (for the UK, Salisbury, 1989; Billinghurst and Whitfield, 1993; Dixon *et al*., 1997; McLean and Sutton, 2005; for Norway, Godager, 2009), other aspects of accessibility such as opening hours (Dixon *et al*., 1997), and the age, gender and ethnicity of doctors (Godager, 2009).^3^ Stated preference studies have shown that, hypothetically, patients are willing to trade‐off measures of consultation quality, thoroughness of physical examinations and the GP's knowledge of the patient against the accessibility of the consultation and waiting times (Vick and Scott, 1998; Scott and Vick, 1999; Cheraghi‐Sohi *et al*., 2008). Revealed preference evidence on the relationship between choice of practice and proxies for quality is more mixed (McLean and Sutton, 2005), though studies from Norway found small positive responses to factors such as practice mortality rates and the volume of services provided (Biorn and Godager, 2010; Iversen and Luras, 2011).

More broadly, our article contributes to the literature on whether choice‐based reforms in public services will provide incentives for firms to increase quality. There has been a great deal of interest in recent years in competition in education (Epple and Romano, 1998; Hoxby, 2000; Epple *et al*., 2004). In this literature, as in health care, the predictions from theoretical models are often ambiguous and the empirical evidence contested (Hoxby, 2000; Bayer and McMillan, 2005; Burgess *et al*., 2005; Rothstein, 2007). Here, we show that quality matters to users of health care when they are choosing an important service provider. Thus a necessary condition for policies that promote choice and competition amongst providers to succeed in improving quality is satisfied in the UK primary health care market.

The article is organised as follows. Section 1 outlines the institutional setting, Section 2 discusses the data, Section 3 our empirical strategy, Section 4 presents the key results, Section 5 exploits these to examine the effect of changes in quality on demand and Section 6 concludes.

## 1 Institutional Setting

The NHS is financed almost entirely from general taxation and patients face no charges for NHS health care apart from a small charge for dispensed medicines. To receive NHS primary care individuals must register with a general (family) practice, which also acts as the gatekeeper for elective (non‐emergency) hospital care. GPs are not employees of the NHS (apart from a small proportion directly employed by Primary Care Trusts (PCTs), the local NHS organisations responsible for the administration of primary care in their area). GPs work in general practices, most of which are limited liability partnerships owned by the GPs. The NHS contracts with the general practices, not with the individual GPs. English practices have on average 4.2 GPs and around 6,600 patients (Information Centre, 2011).

Practice contracts with the NHS to supply services to patients are of two types. Just over half of general practices have the General Medical Services (GMS) contract whose terms are set by national negotiations between the NHS and the British Medical Association (the doctors’ trade union). GMS practices are paid a mixture of lump sums, capitation, quality incentive payments and items of service. Around 75% of practice revenue varies with the number of patients registered with the practice. Over 50% is from capitation payments determined by a national formula which takes account of the demographic mix of practice patients and local morbidity measures. Quality incentives from the Quality and Outcomes Framework (QOF) (Roland, 2004) generate a further 15% of practice revenue. For a given quality level as measured by the QOF score, revenue increases with the number of patients. Practice payments for providing specific services including vaccinating and screening target proportions of the relevant practice population also increase with the total number of patients registered with the practice. Practices are reimbursed for the costs of their premises but have to fund all other expenses, such as hiring practice nurses and clerical staff, from their revenue.

The remaining practices have a Primary Medical Services (PMS) contract which is negotiated between the practice and their local PCT. The practice receives a lump sum for agreeing to provide similar services to those required under the GMS contract, plus additional services for particular patient groups. The amount received is typically what the practice would have received under GMS, plus an addition to cover the cost of the extra services. PMS practices also receive QOF payments, though they are paid less than GMS practices for the same quality achievement because some of the QOF payments relate to activities which are also paid for directly under PMS contracts. As under GMS the practice has to meet its expenses from its revenue. Thus, whether the practice has a GMS or a PMS contract, its total revenue will increase with the number of patients. A rough estimate under the assumption that average revenue and cost per patient are constant is that an additional patient registered with the practice produces revenue of £135, expenses of £80 and net income of £55 per practice partner.^4^

Although practices cannot refuse to accept patients on grounds of race, gender, social class, age, religion, sexual orientation, appearance, disability or medical condition, patients face two constraints when choosing a practice. First, a practice can refuse to accept patients who live outside a catchment area agreed with its PCT. Second, practices can notify their PCT that their list is closed: if this is the case, no new patients will be accepted for a period of between 3 and 12 months. Around 2% of practices have closed lists at any one time.^5^ Practices with closed lists are not eligible for certain types of payments for providing additional services, so that some practices tell potential new patients that they are ‘open but full’ in an attempt to restrict registration. Possibly up to 10% of practices are open but full at any time (National Audit Office, 2008) but since the designation is unofficial and has no legal force its extent and effect on registrations are unclear. We discuss the implications of these restrictions on patient choice of practice in section 4.5.^6^

One of the strands in policy in the English NHS in recent years has been the promotion of competition amongst hospitals and, latterly, amongst general practices (Department of Health, 2010). The national body which controlled entry of new practices was abolished in 2002 and the Department of Health introduced a tendering process to make it easier for new practices to be established, especially in under‐doctored areas (Department of Health, 2006). A website, NHS Choices, has been set up containing information on the characteristics of practices, such as the clinics they offer, their performance under the national quality incentive (QOF) scheme and results from patient satisfaction surveys.^7^ And from 2015 practices will be able to register patients who live outside their catchment area without the obligation to make home visits, thus widening patients’ choice of practice (Mays *et al*., 2014).^8^

## 2 Data

We construct a rich data set on patients and practices by linking NHS administrative data sets (Attribution Data Set (ADS), General Medical Statistics, QOF, Hospital Episode Statistics) with small area census and socio‐economic data from Neighbourhood Statistics. Sources are in Appendix Table C1.

### 2.1 Patients

The ADS contains, for each administratively defined homogenous small geographical area in England (known as a lower super output area, LSOA), the number of patients by age/sex band who are registered with each general practice at 1 April 2010. There are 32,482 LSOAs in England, with a minimum population of 1,000 and a mean population of 1,500.^9^ To reduce the computational burden, we limit our analysis to the choice of practice by patients resident in the East Midlands Strategic Health Authority (SHA). This contains 2,875 LSOAs, has a mixture of densely populated urban areas and rural areas, has a diverse population (so allowing investigation of the effects of ethnicity and other socio‐economic characteristics on tastes for practice characteristics) and it is far from the English‐Welsh and English‐Scottish borders (so we do not have to drop any LSOAs with residents registered in Welsh or Scottish practices whose characteristics we do not observe).^10^ We exclude children because their practice choices are made by their parents and we cannot distinguish in our data between individuals with and without children. We also exclude individuals aged 18–24 because students in post‐high school education may continue to be registered at their parents’ general practice despite living away from home. We therefore analyse the choice of practice by the 3.372 million individuals in the East Midlands SHA who are aged 25 and over.^11^

The ADS data contain age (in bands) and gender of each patient. We attribute socio‐economic characteristics to patients by their LSOA of residence. The characteristics include the proportion of the LSOA who are income deprived (defined as receiving income‐related social security benefits), the proportion of adults with no formal educational qualifications, the proportion who report themselves in fair or good, rather than poor, self‐rated health and the proportion who are of Asian ethnicity. We also categorise an LSOA as urban or rural and by the annual rate of inward migration. These patient and small area‐level variables allow us to examine whether different types of patient have different preferences over practice characteristics.

### 2.2 Practice Characteristics

We use data from the GMS census (taken on 30 September 2010 and 2009) to measure the average age of GPs, the proportion of female GPs and the proportion of GPs qualified in the UK, in Europe, in Asia and elsewhere. We also have data on the type of practice contract (PMS or GMS) and whether the practice has opted out of providing out‐of‐hours care for its patients.^12^ There are no centrally collected practice‐level data on practice catchment areas, or whether practice lists are open, closed or open but full.

### 2.3 Practice Quality

Our measure of practice clinical quality is from the QOF. The QOF is a national pay‐for‐performance scheme introduced in April 2004 and whose broad structure has been maintained subsequently. The quality indicators in the QOF were chosen on the basis of evidence about the effects of the activities they measure on patient health. Higher achievement of the quality indicators has been shown to be associated with fewer emergency hospitalisations for conditions which should be managed in primary care (Dusheiko *et al*., 2011).^13^ From 2006/7 practices could receive up to 1,000 points for achieving quality indicators grouped into four domains (clinical, organisation, patient experience and additional services) and for a holistic care indicator. Each point earned the practice £125.^14^ We use total QOF points for 2006/7. We choose a four‐year lagged measure (choice of practice is observed for 2010) to reduce reverse causality from patient choices to quality.

### 2.4 Distance Measurement and Choice Sets

**Figure 1:**
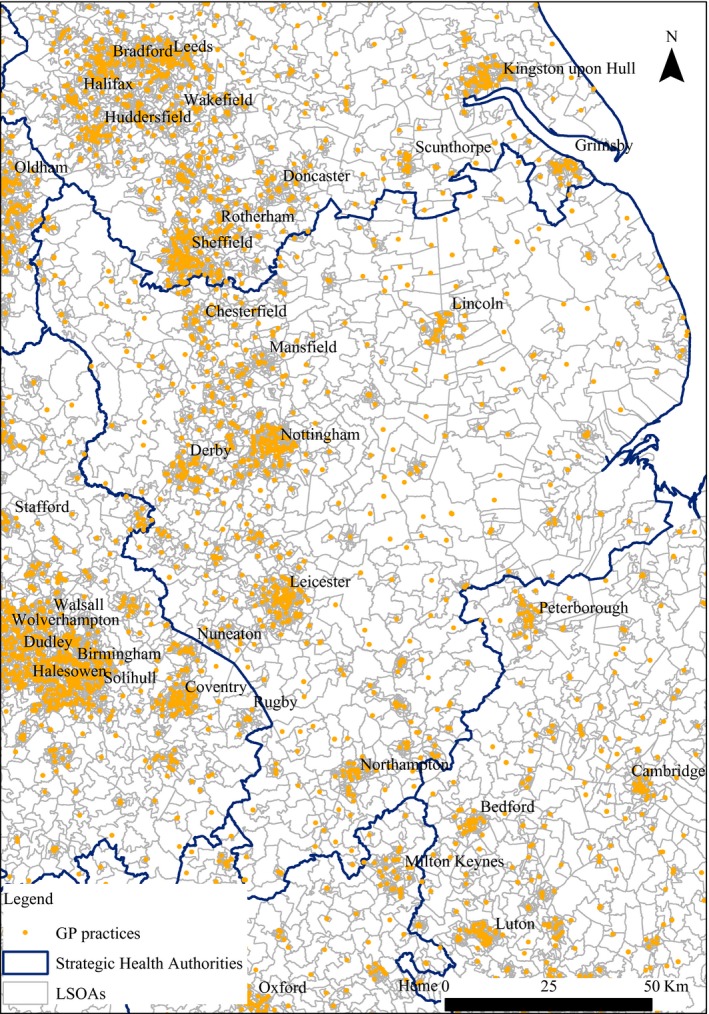
East Midlands Strategic Health Authority: Practice Locations and LSOAs

**Figure 2:**
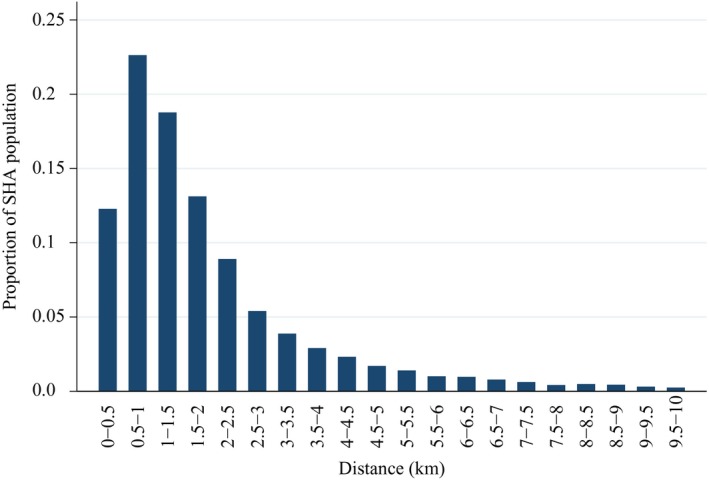
Distribution of Distances to Chosen Practice

Figure 1 shows the practices and LSOAs in the East Midlands. Some practices have more than one surgery (on average each practice had 1.27 surgeries). We calculated the straight line distance between the centroid of each LSOA and all GP surgeries within 50 km of LSOAs in the East Midlands SHA. We use the distance to the nearest branch surgery of a practice from the LSOA centroid as our measure of practice distance. In deciding the appropriate radius for the choice sets facing individuals in LSOAs we make a trade‐off. Setting a wider radius increases the computation burden as more practices are in choice sets but also (see Figure 2) reduces the proportion of the population excluded from the model because their chosen practice is not in the choice set of their LSOA. Based on the data on actual choices, for our baseline model we use a radius of 10 km. This covers the choices of over 99% of the population. Since some urban LSOAs had over 100 practices within 10 km we further restrict the choice set to the 30 practices closest to the centroid of the LSOA. We also estimate models with smaller radii and with different radii for rural and urban LSOAs.^15^

Practices are supervised by administrative bodies known as PCTs. Although patients are not required to register with practices located in the PCT in which they live, they may be less likely to choose practices in a different PCT because PCTs provide information about practices located within the PCT. Moreover, PCT boundaries are in part determined by physical features such as railway lines and rivers which may make it more difficult to access a practice than is suggested by the straight line distance. To allow for this, in estimation we take account of whether practices are in the same PCT as the LSOA of the patient.

### 2.5 Descriptive Statistics

Table 1 presents the practice characteristics, distances and the small area (LSOA) characteristics.^16^ Over a third (37%) of GPs in practices are female and over a quarter (27%) were trained outside Europe.^17^ The mean distance to the nearest practice is 1.2 km and the mean distance to practices within the LSOA choice set is 4.8 km. There are on average 22 practices within the choice set of each LSOA. The mean distance to the chosen practice is 1.9 km and its distribution, shown in Figure 2, is right skewed as 41% of East Midlands SHA patients are registered with the nearest GP practice. Around 25% of practices in LSOA choice sets are located in a different PCT and 16% of patients choose a practice in a different PCT.

**Table 1:** Descriptive Statistics

	Mean	Median	SD	Min	Max	*N*
*GP practice characteristics*						
Average GP age (years) 2009	47.8	46.7	6.7	31.5	72	981
Proportion female GPs 2009	0.365	0.400	0.248	0	1	981
Proportion GPs trained outside Europe 2009	0.265	0.100	0.352	0	1	981
Opted out of out of hours care 2009	0.586	1	0.493	0	1	981
PMS contract 2009	0.483	0	0.500	0	1	981
Dispensing practice 2009	0.207	0	0.405	0	1	981
Patients* aged 25 and over registered with practice	4,902	4,269	3,071	653	24,988	981
*Quality measure*						
QOF 2006/7 total points	955.5	980.1	64.6	426.5	1,000	973
*Average distances from LSOA to practices*						
Distance to practices in LSOA choice set (km)	4.757	4.777	1.727	0.348	9.888	2,875
Distance to chosen practice (km)	1.877	1.480	1.341	0.125	9.867	2,875
Distance to nearest practice (km)	1.197	0.842	1.162	0.023	9.810	2,875
*Practices in different PCT*						
Proportion practices in choice set in different PCT	0.247	0.133	0.273	0	1	2,875
Proportion chosen practices in different PCT	0.160	0	0.231	0	1	2,875
Proportion of nearest practices in different PCT	0.049	0	0.217	0	1	2,875
*LSOA characteristics*						
Income deprivation score	0.143	0.106	0.110	0.013	0.830	2,875
Proportion of adults without qualification	0.231	0.231	0.071	0.035	0.430	2,875
Proportion pop in fair or good self‐rated health	0.907	0.911	0.032	0.760	0.983	2,875
Proportion non white	0.065	0.019	0.013	0	0.095	2,875
Rural	0.269	0	0.444	0	1	2,875
Population inflow 2009/10 (%)	5.07	4.58	2.14	1.72	18.62	2,875
Population growth 2009/10 (%)	0.06	0.07	0.72	−3.44	4.46	2,875
Proportion population registered at nearest practice	0.408	0.350	0.266	0	1	2,875
Number of practices in LSOA choice set	22.4	30	10.3	1	33	2,875

Note

a Whether resident in the East Midlands SHA or outside it.

## 3 Empirical Approach

We estimate conditional logit models of choice of practice. There are *n* ^*A*^ LSOAs and their choice sets contain *n* ^*J*^ different practices in total. All *n*_*a*_ residents in LSOA *a* choose a practice from the same set $C_{a}:n_{a}=\sum_{j\in C_{a}}^{}n_{aj}$, where *n*_*aj*_ is the number of LSOA *a* residents who choose practice *j*. The number of residents choosing practice *j* is $n_{j}=\sum_{a=1}^{n^{A}}n_{aj}$ and there are $N=\sum_{j=1}^{n^{J}}n_{j}=\sum_{a=1}^{n^{A}}n_{a}$ residents in total.

Suppose that utility for individual *i* living in LSOA *a* if he chooses practice *j* is representable by the linear function

(1) $$u_{iaj}=v_{iaj}+\varepsilon_{iaj}={x^{\prime }}_{iaj}\beta +\varepsilon_{iaj},$$

**x**_*iaj*_ **=* *(*x*_1*iaj*_, … , *x*_*Kiaj*_) is a vector of *K* observed variables and *ε*_*iaj*_ is random error term reflecting practice characteristics observed by the individual *i* but not the econometrician. Each resident *i* in LSOA *a* chooses the practice in their choice set *C*_*a*_ which yields the highest realised value of *u*_*iaj*_.

If the *ε*_*iaj*_ errors are independently and identically distributed according to the type 1 extreme value distribution, then (McFadden, 1974) the probability that individual *i* in LSOA *a* chooses practice *j* is

(2) $$P_{iaj}=\exp\left({x^{\prime }}_{iaj}\beta \right){\left[\sum_{\ell \in C_{a}}\exp({x^{\prime }}_{ia\ell }\beta )\right]}^{-1}.$$

If we assume, as in most of our models, that individuals’ preferences over practice characteristics do not vary across different types of individual, only variables which vary by LSOA and practice (**x**_*aj*_) will affect choice probabilities and thus the probability of choice of practice *j* by an individual in LSOA *a* is the same for all individuals in LSOA *a*. Hence,

(3) $$P_{iaj}=P_{aj}=\exp\left({x^{\prime }}_{aj}\beta \right){\left[\sum_{\ell \in C_{a}}\exp({x^{\prime }}_{a\ell }\beta )\right]}^{-1},$$

and the log‐likelihood is

(4) $$\ln\;L=\sum_{a=1}^{n^{A}}\sum_{j^{\prime }\in C_{a}}^{}n_{aj}\ln\left[\frac{\exp({x^{\prime }}_{aj}\beta )}{\sum_{\ell \in C_{a}}\exp({x^{\prime }}_{a\ell }\beta )}\right],$$

so that the log of the choice probability for practice *j* in choice set *C*_*a*_ is weighted by the number of individuals in LSOA *a* who choose practice *j*.

We examine our assumption of homogeneous individual preferences in three ways. First, we estimate separate models, using (4), for each age and gender group, so that *n*_*aj*_ is now the number of residents in an LSOA in a given age/gender band who choose practice *j*. Second, to investigate whether preferences for practices vary with LSOA characteristics, we stratify LSOAs separately by the proportion of the population who are income deprived, who are non‐white, who have no educational qualifications or who are in fair or good self‐reported health. We also estimate separate models based on the amount of inward mobility in the LSOA, on the assumption that LSOAs with more inward mobility will be composed of populations which, on average, have made choices of GP more recently. Third, we allow the coefficients ***β*** in individual utility functions to vary randomly across individuals according to a normal distribution and we estimate mixed logit models of their mean and SD.

## 4 Results

### 4.1 The Effect of Quality, Distance and Practice Characteristics

Table 2 presents our baseline model. Quality is measured by four‐year lagged total QOF points (2006/7) and we allow for non‐linearity of utility in distance with a cubic function of distance from the LSOA centroid to the nearest surgery of the practice. Other covariates are practice characteristics: whether the practice is in the same PCT as the LSOA, mean GP age in months, percentage of female GPs, percentage of GPs qualified outside Europe, whether the practice has a PMS contract and whether the practice has opted‐out of providing out‐of‐hours cover.

**Table 2:** Choice of Practice: Marginal Utility of Quality, Distance, and Practice Characteristics

	Co‐efficient
QOF 2006/7 total points	0.00222***
	(0.00016)
Distance	−0.7512***
	(0.0163)
Practice in different PCT	−0.881***
	(0.044)
Average GP age (months)	−0.00214***
	(0.00014)
Percentage of female GPs	0.00239***
	(0.00034)
Percentage of non‐European qualified GPs	−0.00527***
	(0.00028)
Opted out of 24 hours obligation	0.160***
	(0.037)
PMS contract	0.148***
	(0.033)
BIC	11,597,725
McFadden R^2^	0.3991
*N* LSOA	2,870
*N* practices	973
*N* patients	3,291,581
MRS distance for quality	0.00296***
	(0.00022)

Notes

Conditional logit model of choice of practice by patients aged 25 and over. Model also contained a dummy for missing PMS status. The distance coefficient is ${\hat{\beta }}_{d}+2{\hat{\beta }}_{d^{2}}\overset{¯}{d}+3{\hat{\beta }}_{d^{3}}\bar{d^{2}}$ where $\overset{¯}{d}$ and $\bar{d^{2}}$ are computed as the mean of the LSOA centroid to practice distance in km and the squared distance for the whole sample. MRS is the coefficient on quality divided by the distance coefficient. Standard errors clustered at LSOA level are in parentheses. *p < 0.05, **p < 0.01, ***p < 0.001.

The reported coefficients have the same sign as the effect of an attribute on the probability of choice $\left[\partial {\hat{P}}_{aj}/\partial x_{kaj}^{}={\hat{\beta }}_{k}{\hat{P}}_{aj}\left(1-{\hat{P}}_{aj}\right)\right]$. They estimate the marginal utility from the practice characteristics only up to a positive scalar since $u_{iaj}^{*}=\lambda u_{iaj}^{}=\lambda {x^{\prime }}_{iaj}\beta +\lambda \varepsilon_{iaj}$ yields the same choices as $u_{iaj}$ for all *λ* > 0. Since the scale is determined by the variance of the sample on which the model is estimated, we also report the marginal rate of substitution between practice quality *q*_*j*_ and the distance *d*_*aj*_ in kilometres between the LSOA centroid and the practice. *MRS*_*qd*_ is the additional distance in kilometres that a patient in LSOA *a* would be willing to travel to practice *j* if its quality increased by 1 point. Since it is the ratio of marginal utilities it is invariant with respect to the scale of utility. Thus comparison of the *MRS*_*qd*_ for different samples of patients conveys information about differences in preferences.^18^

Table 2 shows that patients are more likely to choose a practice with higher quality. They dislike distance, preferring practices that are closer to their homes, and, conditional on distance, practices in the same PCT.^19^ In terms of GP characteristics, patients prefer practices with younger GPs, with a higher proportion of female GPs, with a lower proportion of non‐European qualified GPs, practices that have opted out of out‐of‐hours cover and those with PMS contracts. These results for practice gender and ethnicity mix and average age are robust across all estimated models and confirm earlier research findings on the choice of GPs in the UK.^20^

In Appendix A, we examine the robustness of these results to a large set of alternative measures of quality. These include total QOF points for other years, sub‐domains of the QOF, the practice rate of hospital admissions for conditions which ought to be manageable in general practice and patient satisfaction measures based on practice surveys. The results confirm that patients are more likely to choose practices with higher quality. Total QOF points for 2006/7 perform a little better than total points for 2010/11 or the 2006/7–2010/11 average of total points. Many of the other measures of quality are insignificant conditional on inclusion of total 2006/7 QOF points. We therefore retain our baseline single‐quality measure (2006/7 total QOF points) because it is correlated with other measures of quality (see Appendix Table C4), is simpler than models with multiple quality measures and has a better fit than other models with a single quality measure. Total QOF points are also plausible as a measure which affects patient choices since these were publicly reported on the NHS Choices website aimed at informing patient choice.

**Figure 3:**
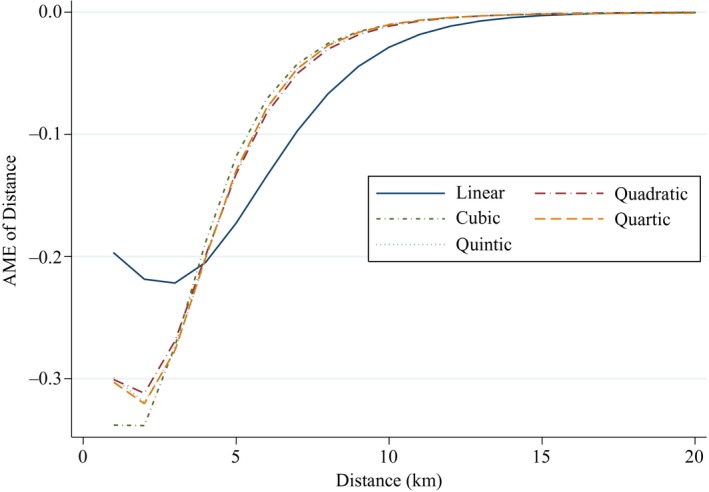
Estimates of the Average Marginal Effects of Distance *Notes*. Plot of ${\left(T\right)}^{-1}\;\sum_{a}\sum_{j\in C_{a}}{\hat{P}}_{aj}\left[\;\;f\left(d_{aj},{\hat{\beta }}_{d}\right),\cdot \right]\;\;\left\{1\;-\;{\hat{P}}_{aj}\left[\;\;f\left(d_{aj},\;{\hat{\beta }}_{d}\right),\;\cdot \right]\right\}\;\;\partial f\left(d_{aj},\;{\hat{\beta }}_{d}\right)/\partial d_{ah}$ where ${\hat{P}}_{aj}\left[f\left(d_{aj},{\hat{\beta }}_{d}\right),\cdot \right]$ is the predicted probability of choice of practice evaluated at the mean of quality and of the non‐distance explanatories, *T* is the number of LSOA‐practice combinations and $f(d_{aj},{\hat{\beta }}_{d})$ is a linear, quadratic, cubic, quartic or quintic function of distance. ${\hat{P}}_{aj}\left[f\left(d_{aj},{\hat{\beta }}_{d}\right),\cdot \right]$ is derived from models with same quality measure and covariates as Table 2.

Because of the importance of distance in determining practice choice we investigated the robustness of the baseline model to alternative polynomial functions (linear to quintic) of distance. Figure 3 plots the average marginal effect of distance on the probability of choice of practice and shows that the negative marginal effects of distance decrease with distance in all the specifications. The effect of quality is positive and significant in all specifications. Including squared and cubed distance reduces the coefficient on quality slightly but further addition of fourth and fifth powers leaves the quality coefficient unchanged and renders some of the powers of distance statistically insignificant.^21^ We therefore use the cubic specification as our baseline model.

### 4.2 Is There Heterogeneity in Patient Preferences?

We begin by examining observed heterogeneity by age and gender. Previous literature has suggested that preferences for medical practitioners differ across men and women and individuals of different ages. We estimate separate models for 12 age and gender groups, using the same specification as the baseline model. We report the quality and distance coefficients in Table 3. Preferences for quality and distance appear to be non‐linear in age. Women in the middle age groups have larger quality coefficients and less negative distance coefficients. While differences in coefficients across age groups may be due to differences in the scale on which utility is measured for different age groups, the ratio of the marginal utilities on quality and distance is invariant to scaling and so differences in the MRS reflect differences in preferences across age groups. The MRS estimates show that middle‐aged women place a higher value on quality relative to the distance they are willing to travel to a practice. Men seem to have more homogeneous preferences with the exception of the youngest age (25–34) group. This group have both the smallest marginal utility from quality coefficient across all age and gender groups and by far the lowest willingness to travel for higher quality. Men in this age are the lowest users of GP care (Hippisley‐Cox *et al*., 2007) and therefore may not place a high value on quality.

**Table 3:** Comparison of Practice Choice Models by Age and Gender groups

	All	Female all	F24–34	F35–44	F45–64	F65–74	F75plus
QOF 2006/7 total points	0.00222***	0.00236***	0.00208***	0.00257***	0.00251***	0.00228***	0.00216***
	(0.00016)	(0.00016)	(0.00016)	(0.00017)	(0.00017)	(0.00019)	(0.00021)
Distance	−0.7512***	−0.7575***	−0.7708***	−0.7499***	−0.7433***	−0.7679***	−0.8182***
	(0.0163)	(0.0165)	(0.0175)	(0.0165)	(0.0169)	(0.0178)	(0.0198)
MRS	0.00296***	0.00312***	0.00270***	0.00342***	0.00338***	0.00297***	0.00264***
	(0.00022)	(0.00023)	(0.00022)	(0.00024)	(0.00025)	(0.00026)	(0.00026)
BIC	11,597,725	5,769,627	1,054,605	1,179,122	2,110,832	719,211	701,624
McFadden R^2^	0.3991	0.4059	0.4005	0.4040	0.3995	0.4107	0.4336
*N* patients	3,350,561	1,688,960	295,329	341,336	616,961	217,072	218,176

	Male all	M25–34	M35–44	M45–64	M64–74	M75plus
QOF 2006/7 total points	0.00208***	0.00137***	0.00219***	0.00234***	0.00214***	0.00234***
	(0.00015)	(0.00018)	(0.00016)	(0.00017)	(0.00019)	(0.0002)
Distance	−0.7447***	−0.7550***	−0.7530***	−0.7396***	−0.7426***	−0.7843***
	(0.0161)	(0.0178)	(0.0162)	(0.0164)	(0.0176)	(0.0184)
MRS	0.00279***	0.00181***	0.00291***	0.00316***	0.00289***	0.00298***
	(0.00021)	(0.00024)	(0.00022)	(0.00023)	(0.00027)	(0.00026)
BIC	5,827,061	1,144,601	1,270,483	2,215,871	696,124	495,759
McFadden R^2^	0.3924	0.3824	0.3933	0.3907	0.4001	0.4139
*N* patients	1,661,426	309,837	358,003	636,112	207,247	150,139

Notes

Conditional logit models. All models contain same covariates as model in Table 2. Distance coefficient is ${\hat{\beta }}_{d}+2{\hat{\beta }}_{d^{2}}\overset{¯}{d}+3{\hat{\beta }}_{d^{3}}\bar{d^{2}}$ where $\overset{¯}{d}$ and $\bar{d^{2}}$ are computed as the mean of the LSOA centroid to practice distance and squared distance for the whole sample. MRS is the coefficient on quality divided by the distance coefficient. N LSOAs: 2,870. N practices: 973. Standard errors clustered at LSOA level are in parentheses. *p < 0.05, **p < 0.01, ***p < 0.001.

Table 4 allows for heterogeneity of preferences between individuals resident in different types of small area. In all cases patients are more likely to choose practices which have higher quality and are closer, but there are some differences across small area characteristics. Residents of rural areas have a slightly higher MRS of quality for distance to those in urban areas. Individuals in LSOAs with fewer income‐deprived residents and those in LSOAs with better educated populations have substantially higher MRS of quality against distance compared with those in more deprived or worse educated LSOAs. Residents in LSOAs with healthier populations place a higher value on quality relative to distance than those in LSOAs with less healthy populations. Those in LSOAs with a higher Asian population proportion place a higher value on quality relative to distance than those in LSOAs with a smaller Asian population proportion. Finally, residents in LSOAs with a high population inflow have higher MRS between quality and distance than those in low inflow LSOAs.

**Table 4:** Choice Models for LSOA Samples Stratified by Socio‐economic Characteristics and Population Mobility

	Urban	Rural	Lower income deprivation	Higher income deprivation	Better education	Worse education
QOF 2006/7 total points	0.00224***	0.00188***	0.00249***	0.00144***	0.00238***	0.00181***
	(0.00014)	(0.00046)	(0.00019)	(0.00026)	(0.00019)	(0.00028)
Distance	−0.8730***	−0.6706***	−0.7714***	−0.7755***	−0.7450***	−0.8383***
	(0.0172)	(0.0263)	(0.0175)	(0.0350)	(0.0179)	(0.0320)
MRS	0.00256***	0.00281***	0.00323***	0.00186***	0.00320***	0.00216***
	(0.00017)	(0.00070)	(0.00026)	(0.00034)	(0.00026)	(0.00033)
BIC	9,327,639	2,243,349	8,954,600	2,610,534	9,289,002	2,295,492
McFadden R^2^	0.3699	0.5014	0.4138	0.3515	0.3997	0.4001
*N* LSOA	2,100	770	2,295	575	2,295	575
*N* practices	796	854	971	672	968	738
*N* patients	2,404,472	946,089	2,711,039	639,522	2,701,394	649,167

	Better health	Worse health	Less Asian	More Asian	Less population inflow	More population inflow
QOF 2006/7 total points	0.00253***	0.00139***	0.00213***	0.00240***	0.00218***	0.00248***
	(0.00018)	(0.00029)	(0.00020)	(0.00020)	(0.00019)	(0.00025)
Distance	−0.7360***	−0.8782***	−0.7787***	−0.7607***	−0.7789***	−0.6482
	(0.0178)	(0.0340)	(0.0179)	(0.0319)	(0.0178)	0.0367
MRS	0.00343***	0.00158***	0.00274***	0.00315***	0.0028***	0.00382***
	(0.00026)	(0.00034)	(0.00027)	(0.00029)	(0.00025)	(0.00045)
BIC	9,226,183	2,362,093	8,421,525	3,148,128	8,897,398	2,685,028
McFadden R^2^	0.3967	0.4109	0.4322	0.2955	0.4217	0.3142
*N* LSOA	2,296	574	2,294	576	2,295	575
*N* practices	966	765	970	583	973	644
*N* patients	2,696,060	654,501	2,682,712	667,849	2,705,051	645,510

Notes

Conditional logit models of practice choice for patients 24 + . All models contain same covariates as model in Table 2. Distance coefficient is ${\hat{\beta }}_{d}+2{\hat{\beta }}_{d^{2}}\overset{¯}{d}+3{\hat{\beta }}_{d^{3}}\bar{d^{2}}$ where $\overset{¯}{d}$ and $\bar{d^{2}}$ are computed as the mean of the LSOA centroid to practice distance and squared distance for the full sample of 2,870 LSOAs. MRS is the coefficient on quality divided by the distance coefficient. Urrepoban LSOAs are LSOAs classified as Urban or Town and Rural LSOAs are those classified as Village or Hamlet and Isolated Dwelling. Lower inc depriv: LSOAs in bottom 4 quintiles of income deprivation. Higher inc depriv: LSOAs in top quintile of income deprivation. Better education: LSOAs in bottom 4 quintiles of proportion of population with no formal educational qualifications. Less Educ: LSOAs in top quintile of educational deprivation. Better health: LSOAs in top four quintiles of proportion reporting being in good or fair health. Worse Health: LSOAs in bottom quintile of proportion reporting being in good or fair health. Less Asian: LSOAs in bottom four quintiles of proportion of LSOA population classified as Asian or Asian British. More Asian: LSOAs in top quintile of LSOA population classified as Asian or British Asian. Less inflow: LSOAs in bottom four quintiles of proportionate population inflow distribution for 2009/10. More inflow: LSOAs in top quintile of inflow distribution. Standard errors clustered at LSOA level are in parentheses. *p < 0.05, **p < 0.01, ***p < 0.001.

These differences in the weight that individuals in different types of LSOA place on quality when choosing a practice seem plausible. Individuals in rural areas expect to travel further for all types of services, so they are more willing to travel for a practice with higher quality than patients in urban areas. Residents in LSOAs with fewer income‐deprived individuals may be more able to take time off work without incurring a financial penalty and so be less concerned about the travel time required to use their GP than residents in LSOAs with higher income deprivation. Residents of LSOAs with better educated populations may be better able to find, and interpret, measures of practice quality. Our finding that patients in LSOAs with healthier populations place a higher value on quality relative to distance than those in LSOAs with less healthy populations again supports a travel cost story: less healthy patients want to be closer to their practice because they expect to visit it more frequently. The fact that Asian or British Asian residents on average have more morbidity for many conditions treatable in primary care such as diabetes and heart disease (Scarborough *et al*., 2010) may explain why residents in LSOAs with a higher proportion of Asian or British Asian population place a greater weight on quality. New residents are more likely to have chosen their practices more recently and will – unlike earlier choosers of a practice – have had access to the publicly available QOF scores when making their choice. Thus, new arrivals in an area might be expected to have better information about quality than established residents and so small areas characterised by more new arrivals (as measured by the inflow rate) will give greater weight to quality.^22^

The models in Tables 3 and 4 allow for observed heterogeneity. Table 5 compares the results from a mixed logit model, which allows for unobserved heterogeneity, with those from our baseline conditional logit specification. SDs of the mixed logit coefficients are not significantly different from zero except for the distance and quality variables. The mean mixed logit model coefficients on quality and distance are larger than those from the conditional logit model. The mixed logit MRS between distance and quality shows the distance an individual with average preferences would be willing to travel for an additional QOF point. This is only a little greater than the estimate from the conditional logit model (3.5 *versus* 3.0 metres). We, therefore, feel that the estimates from the simpler conditional logit baseline model are a reasonable representation of average preferences over practice characteristics.

**Table 5:** Choice Model: Mixed and Conditional Logit Specification

	Mixed logit	Conditional logit
	Mean of coefficients	Co‐efficient
QOF 2006/7 total points	0.00294***	0.00222***
	(0.00027)	(0.00016)
Distance km	−1.596***	−1.606***
	(0.04167)	(0.04044)
Distance squared km	0.113***	0.126***
	(0.01238)	(0.01189)
Distance cubic km	−0.00419***	−0.00447***
	(0.00093)	(0.00091)
Practice in different PCT	−0.923***	−0.881***
	(0.07095)	(0.04436)
Average GP age (months)	−0.00217***	−0.00214***
	(0.00014)	(0.00014)
Percentage of female GPs	0.00236***	0.00239***
	(0.000341)	(0.000336)
Percentage of non‐European qualified GPs	−0.00521***	−0.00527***
	(0.000285)	(0.000281)
Opted out of 24 hours obligation	0.167***	0.160***
	(0.0373)	(0.03748)
PMS contract	0.154***	0.148***
	(0.03322)	(0.03317)
Distance	−0.784***	−0.751***
	(0.018)	(0.016)
MRS distance for quality	0.00352***	0.00296***
	(0.00033)	(0.00022)
	SD of coefficients	
QOF 2006/7 total points	0.00333***	
	(0.00049)	
Distance km	0.212***	
	(0.02589)	
Distance squared km	0.0000127	
	(0.00386)	
Distance cubic km	0.000261	
	(0.00028)	
Practice in different PCT	0.368	
	(0.25843)	
Average GP age (months)	0.00040	
	(0.00049)	
Percentage of female GPs	0.000192	
	(0.000352)	
Percentage of non‐European qualified GPs	0.000971	
	(0.000595)	
Opted out of 24 hours obligation	0.253	
	(0.1478)	
PMS contract	0.0525	
	(0.2927)	
BIC	11,587,409	11,597,725

Notes

For the mixed and conditional logit models the distance coefficient is ${\hat{\beta }}_{d}+2{\hat{\beta }}_{d^{2}}\overset{¯}{d}+3{\hat{\beta }}_{d^{3}}\bar{d^{2}}$ where $\overset{¯}{d}$ and $\bar{d^{2}}$ are computed as the mean of the LSOA centroid to practice distance and squared distance for the whole sample. Mixed logit model estimated with Stata *mixlogit*. MRS is the coefficient on quality divided by the coefficient on distance. For the mixed logit the mean coefficients are used. Delta method (*nlcom*) used to compute standard errors on the MRS. Both models have 3,335,061 patients, 2,870 LSOAs, 973 practices. Both models also contain a dummy for practices with missing PMS status. Standard errors clustered at LSOA level are in parentheses. *p < 0.05, **p < 0.01, ***p < 0.001.

### 4.3 Robustness to Specification of the Choice Set: Catchment Areas and Closed Lists

In interpreting the results, we assume that practice lists reflect patient preferences rather than practice decisions. But, as noted in Section 1, there are two potential constraints on patient choice. First, practices can agree a catchment area with their PCT and are not obliged to accept patients living outside this catchment area. If we specify LSOA choice sets with a radius greater than that of practice catchment areas, then the estimated negative effect of distance will overstate the effect of distance on patient choices. But if our assumed choice set radii are smaller than practice catchment areas radii, the estimated model coefficients will be consistent, given the assumed preferences and error distribution, since we are observing patient rather than practice choices within a choice set which is independent of patient choices and therefore satisfies the uniform conditioning property of McFadden (1978). Further, the ability of practices to set catchment areas will not in itself produce an association between the proportion of an LSOA's patients choosing a practice and the practice's quality. It is possible that a practice whose patients are closer to the practice will have higher quality, either because it is harder to achieve higher quality if there is less contact between patients and GPs or because practices with higher quality have higher demand from patients and ration demand by setting smaller catchment areas for any given list size desired by the practice. Either of these mechanisms would lead to a negative association between practice quality and the average distance from the practice of the practice's patients from all LSOAs in its catchment area. But it would not imply any relationship between practice quality and the proportion of patients in any particular LSOA choosing the practice.

The second constraint on patient choice of practice is that practices can, with the agreement of their PCT, formally close their lists to new patients for between 3 and 12 months at a time. Some practices also declare informally that their lists are ‘open but full’ and they are not accepting new patients. However, for practices intending to stay in business, list closures must be temporary since each year around 8% of patients will leave a practice list (primarily because of residential moves) (Hippisley‐Cox *et al*., 2005). At any time around 2% of practices have formally closed lists and possibly up to 10% have open but full lists. Given this, list closure only affects the choices of a small minority of patients. So we think our estimates are unlikely to be substantially biased by catchment areas or temporary list closure.

However, to examine these concerns we undertake two robustness tests. The first examines the effect of both catchment areas and list closure. We examine the sensitivity of the estimates of the effects of distance, quality and other practice characteristics to our specification of choice set radius. Since practices can ration demand by restricting their catchment areas and by temporary list closure it is less likely that practices with smaller catchment areas are also closing their lists. We therefore estimate models in which the radius of the choice set for LSOAs is restricted progressively from 10 km (our baseline model) down to 8 km, 6 km, 4 km and 2 km, which allows us to compare estimates across radii.^23^ Second, to examine the effect of list closure, we estimate separate models for LSOAs in the top quintile and bottom four quintiles of population growth. The faster the population in an area is growing, the more likely is it that there will be excess demand and more frequent recourse to closed lists.

A finding of little difference in estimates across models suggests that our results reflect patient choices and preferences rather than GP rationing of demand via catchment areas and list closure. Table 6 presents these estimates. Columns (1)–(5) show that the coefficient on quality is remarkably stable across models with different choice set radii but the MRS of distance for quality falls as the choice set radius shrinks because the distance coefficient increases.^24^ This is in line with Figure 3 which shows the effect of distance from the cubic model with a 10 km choice set radius declines with distance (see Appendix Table C11 for the full results). In column (6), we allow for different choice sets across rural and urban residents. We estimate a model with larger (7 km) choice sets for rural LSOAs than for urban LSOAs (3 km).^25^ The quality coefficient is similar to those in the other models and the MRS between quality and distance is greater than in our baseline 10 km choice model because the effect of distance is greater. Column (7) shows the results for slower growing LSOAs. These are very similar to the baseline 10 km choice model. While faster growing LSOAs are more likely to have practices attempting to ration demand and so have smaller catchment areas and more temporary list closures, column (8) shows that quality still has a significant effect on individual demand in these areas. In fact the coefficient on quality is somewhat greater than its estimated effect in the baseline model, giving a higher MRS between quality and distance where population inflow is higher. This perhaps reflects the fact that in such areas there are more patients who have made choices more recently.

**Table 6:** Choice Models with Different Choice Set Radii and Stratified by LSOA Population Growth

	(1)	(2)	(3)	(4)	(5)	(6)	(7)	(8)
	Choice set radius						Population growth	
	10 km	8 km	6 km	4 km	2 km	Urban 3 km Rural 7 km	Lower 4 quintiles	Top quintile
QOF 2006/7 total points	0.00222***	0.00217***	0.00217***	0.00230***	0.00215***	0.00219***	0.00208***	0.00285***
	(0.00016)	(0.00015)	(0.00015)	(0.00016)	(0.00018)	(0.00016)	(0.00017)	(0.00042)
Distance	−0.7512***	−0.7717***	−0.7457***	−0.6715***	−2.0356	−0.6631***	−0.7506***	−0.7554***
	(0.0163)	(0.0167)	(0.0273)	(0.1728)	(2.9421)	(0.0255)	(0.0184	(0.0337)
MRS	0.00296***	0.00281***	0.00291***	0.00342***	−0.00106	0.00331***	0.00277***	0.00377***
	(0.00022)	(0.00021)	(0.00023)	(0.00091)	(0.00153)	(0.00027)	(0.00023	(0.00058)
McFadden R^2^	0.3991	0.3665	0.3151	0.2345	0.1081	0.2355	0.3938	0.4224
N LSOA	2,870	2,806	2,670	2,428	1,925	2,676	2,296	574
*N* practices	973	923	853	738	605	836	945	772
*N* patients	3,291,581	3,190,284	3,003,036	2,661,832	1,844,207	2,773,892	2,680,561	670,000
Percentage of East Midlands population choosing practice outside this choice set	0.21	1.69	4.51	11.00	32.35	8.56		

Notes

Conditional logit models of choice of practice. Choice sets are all practices within stated distance of LSOA centroid and, if there are more than 30 such, the 30 nearest to the LSOA centroid. Choice set for the models stratified by LSOA population growth is all practice within 10 km, and if there are more than 30 such, the 30 nearest to the LSOA centroid. All models have the same covariates and cubic distance specification as Table 2. The distance coefficient is ${\hat{\beta }}_{d}+2{\hat{\beta }}_{d^{2}}\overset{¯}{d}+3{\hat{\beta }}_{d^{3}}\bar{d^{2}}$ where $\overset{¯}{d}$ and $\bar{d^{2}}$ are computed as the mean of the LSOA centroid to practice distance and squared distance for the sample with 10 km radius choice set for all models to ensure that the computed distance coefficient varies across models only because of variation in the linear, quadratic and cubic distance coefficients. MRS is the quality coefficient divided by the distance coefficient. Standard errors clustered at LSOA level are in parentheses. *p* *<* *0.05, **p* *<* *0.01, ***p* *<* *0.001.

In sum, the similarity of the estimates across the models in Table 6 suggests that the choice process is similar across the choice sets. We conclude that our estimates are robust to potential GP ability to set catchment areas or temporarily closed lists. The results also suggest that catchment areas are not constraining patients’ choice amongst practices.^26^

### 4.4 Endogeneity of Quality

It is possible that practice quality is determined in part by the demographic, socio‐economic and health characteristics of the patients on its list. For example, some patient population may make it easier for practices to achieve their QOF targets. Further, if different patient types have different preferences over practice characteristics, the quality measure may be correlated with unobserved demand factors. Endogeneity bias could go either way: practices which are better could attract more complex patients with whom it is more difficult to achieve QOF points, or better educated individuals who may be easier to treat may be more likely to choose better practices. Our use of a lagged measure of practice quality should reduce this source of endogeneity bias. However, measurement error and unobserved practice characteristics affecting demand and correlated with quality may also contribute to endogeneity.

To allow for possible endogeneity from all sources, we estimate a model in which we instrument practice quality. To motivate our choice of instrument, we use the fact that PCTs had clinical governance responsibility for their practices. They monitored the prescribing of their practices, inspected their premises, audited their QOF returns, provided financial assistance for practice computing and financed community nurses to complement GP services. They also provided information to practices comparing their performance with other practices in the PCT. All these activities were intended to increase quality of care in their area and so are likely to affect the QOF performance of all their practices. This suggests that the average quality of all other practices in the PCT is likely to be a good predictor of a practice's quality. We therefore use this instrument to examine the possible bias in our estimates due to endogeneity.

We implement our IV estimates using two‐stage residual inclusion (Terza *et al*., 2008). We first estimate an OLS model of practice quality for all practices in the choice sets of LSOAs in the East Midlands. In addition to the instrument, the first‐stage quality model contains the variables in the choice model, averaged over the LSOAs whose choice sets contain the practice. The residuals from the first stage model are included in the second stage conditional logit model as an additional explanatory variable. The estimated coefficient on the quality measure in the choice model is an unbiased estimate of the effect of quality if the instrument is valid. We bootstrap the standard errors on the coefficients in the second stage choice model.^27^ The upper part of Table 7 reports the first stage results. The IV for quality has an F‐statistic of 16.53, comfortably greater than the conventional critical value of 10 (Stock *et al*., 2002). The lower part of Table 7 presents the second stage. The coefficient on instrumented quality is around three times as large as on un‐instrumented quality and, since the coefficient on distance is almost unchanged, the MRS is also three times as large. However, the quality residuals are not significant in this 2SRI second stage (p = 0.057), so we cannot reject the null of no endogeneity. Given these results from the instrumented quality model, we prefer to use the more conservative estimates from the non‐instrumented model. These will, if anything, underestimate the effects of quality on demand.^28^

**Table 7:** Choice Model with Instrumented General Practice Quality

First stage OLS model of practice quality	
	Co‐efficient
IV: Mean quality all other practices in PCT	0.530***
	(0.130)
Different PCT	9.672
	(6.048)
Distance	−36.23
	(919.393)
Distance squared	5.668
	(3.57)
Distance cubed	−0.253
	(0.205)
Av GP age months	−0.1045**
	(0.0350)
Percentage of female GPs	0.1295
	(0.0950)
Percentage of non‐European qualified GPs	0.1927**
	(0.07600)
Opted out	11.34*
	(4.75)
PMS contract	10.08
	(5.25)
Constant	552.4***
	(128.8)
F test on IV	16.53

Second stage: choice of practice (conditional logit)				
	Full sample model coefficient	Full sample model SE	Mean coefficient bootstrap samples	SD of coefficient in bootstrap samples
QOF 2006/7 total points	0.00622**	(0.00211)	0.00615	(0.00215)
1st stage residual QOF 2006/7	−0.00398	(0.00209)	−0.00390	(0.00212)
Distance	−0.7517***	(0.0163)	−0.75071	(0.01602)
Practice in different PCT	−0.882***	(0.044)	−0.87626	(0.03927)
Average GP age (months)	−0.00175***	(0.00025)	−0.00172	(0.00027)
Percentage of female GPs	0.00194***	(0.00041)	0.00199	(0.00035)
Percentage of non‐European qualified GPs	−0.00448***	(0.00051)	−0.00450	(0.00053)
Opted out of 24 hours obligation	0.115**	(0.043)	0.10720	(0.04163)
PMS contract	0.106**	(0.038)	0.09971	(0.03821)
BIC	11,596,983		38,756,048	225,710
McFadden R^2^	0.3992		0.39903	0.00404
MRS	0.0083**	(0.0028)	0.00821	0.00288

Notes

First stage model: practice QOF 2006/7 points regressed on mean of QOF 2006/7 points of all other practices in the same PCT and on means of all other explanatories in practice choice model taken over all LSOAs whose choice set includes the practice, with SEs clustered on PCTs. Second stage models: conditional logit models of practice choice with all explanatories from model in Table 2 plus quality residuals from first stage model. For the second stage practice choice models, we report the coefficients and SEs from the 2SRI model estimated with the full sample of LSOAs, the average of the coefficients estimated in the bootstrap samples and the SD of the coefficient from bootstrap model. The coefficient on distance is ${\hat{\beta }}_{d}+2{\hat{\beta }}_{d^{2}}\overset{¯}{d}+3{\hat{\beta }}_{d^{3}}\bar{d^{2}}$ where $\overset{¯}{d}$ and $\bar{d^{2}}$ are computed as the mean of the LSOA centroid to practice distance and squared distance for the whole sample. MRS is the coefficient on distance divided by the coefficient on quality. Second stage standard errors are clustered at LSOA level. *p < 0.05, **p < 0.01, ***p < 0.001.

## 5 The Estimated Effect of Quality on Demand

We use the results from the baseline model of Table 2 to illustrate the importance of quality for patients and practices. First, we examine how much further individuals will be willing to travel for a 1 SD change in quality and how this compares to their willingness to trade distance for other practice characteristics (e.g. the percentage of female GPs). Second, we examine one aspect of practices’ incentives to increase their quality by estimating how many patients a practice will gain from a 1 SD increase in its quality. Third, we investigate the extent to which practices will lose patients when a rival increases its quality. If practices gain a large number of patients when their quality increases or lose them when rivals increase their quality, then relaxing constraints on choice is likely to increase GPs’ incentives to provide higher quality.

Table 8 summarises the estimated effect of quality and other practice characteristics on individual's practice choice in a number of different metrics. Columns (1)–(3) examine individual level preferences for a characteristic with respect to distance. The first column re‐reports the coefficients from the baseline model. These show the marginal utility (up to a linear transformation) from a one‐unit increase in practice characteristics. QOF quality is measured as points, average GP age in months, female and non‐European qualified GPs are measured as percentages, rather than as proportions. Column (2) shows the number of additional metres a patient would be willing to travel to a practice if each practice characteristic increased by one unit. As the different characteristics are measured in different units, column (3) reports the number of additional metres an individual would be willing to travel if a characteristic increased by 1 SD.

**Table 8:** Effect Sizes for Quality and Other Practice Characteristics

	(1)	(2)	(3)	(4)	(5)	(6)
	Co‐efficient	Extra metres for 1 unit increase	Extra metres for 1 SD increase	Average marginal effect	Patients gained from 1 SD increase	Percentage increase in practice list from 1 SD increase
QOF total points 2006/7	0.00222	2.96	191	0.0000786	917	16.48
	(0.00016)	(0.22)	(14)	(0.0000129)	(94)	(1.69)
Average GP age (months)	−0.00214	−2.85	−229	−0.0000758	−1,002	−18.01
	(0.00014)	(0.19)	(15)	0.0000062)	(51)	(0.92)
% female GPs	0.00239	3.18	79	0.0000845	351	6.31
	(0.00034)	(0.45)	(11)	(0.0000130)	(34)	(0.61)
% non‐Europ qualif GPs	−0.00527	−7.02	−247	−0.0001867	−1,085	−19.50
	(0.00028)	(0.39)	(14)	(0.0000192)	(70)	(1.26)

Notes

Extra metres: number of extra metres patients would be willing to travel for practice characteristic greater by 1 unit (column (2)) or 1 SD units (column (3)) increase in characteristic. Average marginal effect: average change in probability of patients from an LSOA choosing a practice from 1 unit increase in characteristic (1 QOF point, 1 month of average GP age, 1% female and non‐European qualified GPs). Patients gained: number of additional patients aged 25 and over choosing a practice if characteristic increased by 1 SD. Average marginal effects, average estimated patients gained and % increase in list are computed for the 415 practices which have at least 99% of their patients from LSOAs in the East Midlands. Standard errors are in parentheses. All coefficients and effects are significant at 0.1%. See Appendix B for details of calculations.

As the average individual chooses a practice just under 2 km away, our results show they would be willing to travel about 10% further if a practice had a 1 SD increase (65 QOF points) in quality. The absolute values of the effects of a 1 SD increase are roughly of the same order of magnitude for quality, the average age of GPs and the percentage of non‐European qualified GPs. By contrast, increasing the percentage of female GPs by 1 SD (25%, equivalent to replacing a male GP with a female GP in a four‐GP practice) has a much smaller effect on individuals’ willingness to travel further to a practice.

Columns (4)–(6) examine the estimated effect on practices. Column (4) reports the average marginal effects on the probability that a patient will choose a practice. These are tiny but what matters for the incentive for practices to increase quality is the number of patients they will gain. This depends both on the (small) effect of quality on the probability of any one patient choosing the practice and the (large) number of patients in whose choice set the practice lies and whose probabilities of choosing the practice are increased. An average practice has 74,529 potential patients aged 25 and over resident within 5 km and 25,070 within 2 km. Column (5) shows that the estimated increase in number of patients in a practice from a 1 SD increase in quality is 917. For comparison, the absolute values of the change in the number of patients from a 1 SD (80 months) increase in GP average age and in the percentage of non‐European qualified GPs (35%) are similar to those for quality, whilst the increase in patient numbers from a 1 SD increase in the percentage of female GPs is about half the size of that of a 1 SD change in quality.^29^

Column (6) presents these increases as a proportion of the average number of patients aged 25 and over registered with the practice. This shows the change in patient numbers from a change in quality is important relative to the average number of patients aged 25 and over at just under 17%.^30^ It should be noted that these estimates are the long‐run effects since our estimates are for a stock of patients and average list turnover is around 8% per annum. The short‐term effect of changes in quality (and other practice characteristic changes) will therefore be smaller.

We also examine practice cross‐quality elasticities of demand to estimate how many patients a practice will lose if one of its rivals increases its quality. (Details of the computations are in Appendix B.) The larger the cross‐quality elasticities, the more likely is it that qualities are strategic substitutes and that in the long run the equilibrium quality will rise as other practices will respond by increasing their quality thereby increasing equilibrium quality. Here we only estimate the short‐run responses, that is we assume that practices do not respond if they lose patients.

**Figure 4:**
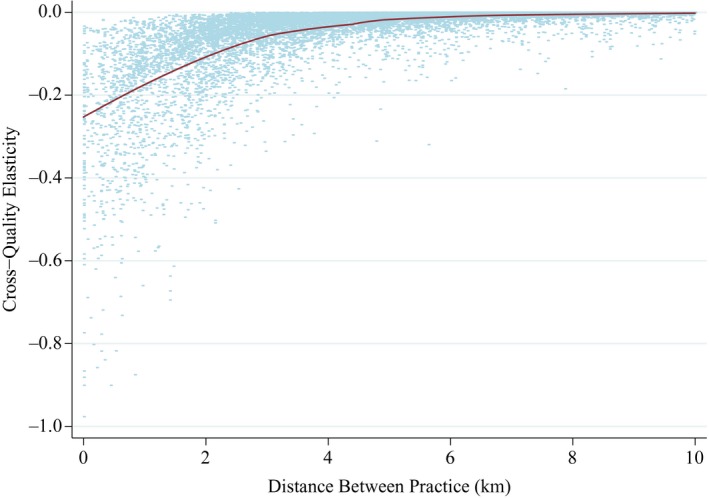
Plot of Cross Practice Quality Elasticities against Distance to Other Practice *Notes*. Cross‐quality elasticity: % change in predicted number of patients in a practice resulting from a 1% increase in quality (total QOF points 2006/7) of a rival practice. Two practices are rivals if there is at least one LSOA choice set of which they are both members. Predictions are from the coefficients from the model in Table 2 applied to practices which draw at least 99% of their list from the East Midlands. The line is a loess regression of elasticity on distance.

The average cross‐practice quality elasticity is −0.044. But there is considerable variation in the cross‐quality elasticities and some cross‐quality elasticities are relatively large. Figure 4 plots the cross‐practice quality elasticities against distance to the rival practice whose quality has increased. While there is variation in cross‐elasticities at any given inter‐practice distance, the plotted loess regression line shows that on average cross‐elasticities decline with the distance between practices. The mean cross‐quality elasticity of the average 14.7 practices within 4 km is −0.15, while the average 2.8 rivals within 2 km have a mean cross‐quality elasticity of −0.25. At longer distances, cross‐quality elasticities are essentially zero. Thus, our estimates suggest that practices operate in geographically small markets, so close practices are substitutes while those further away are not.

**Figure 5:**
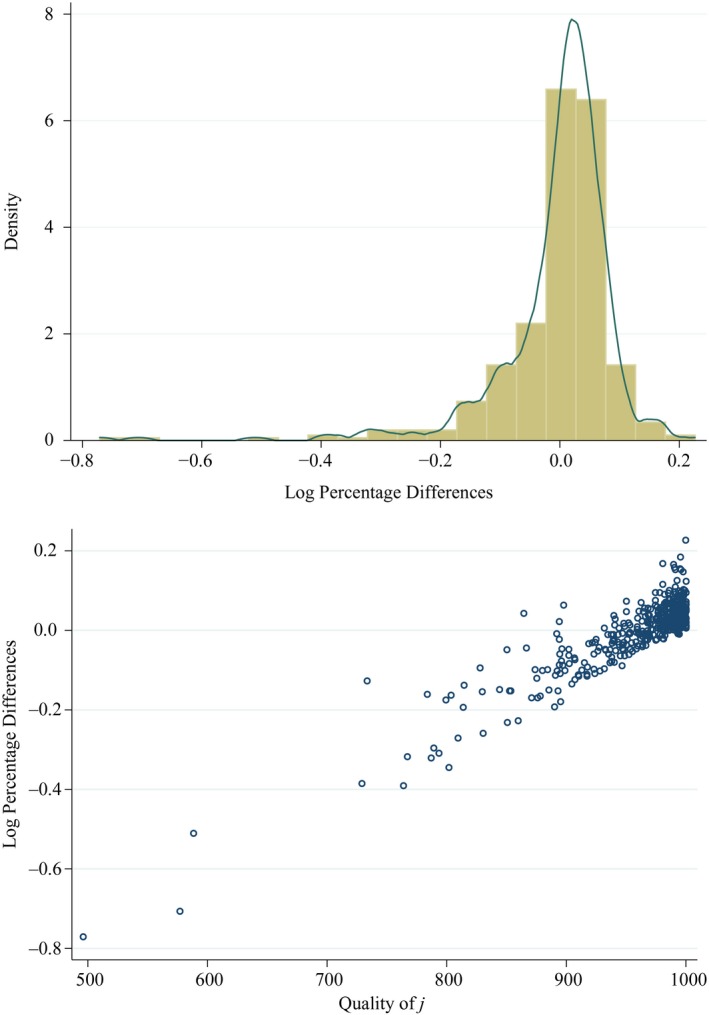
Effect of Quality Information on Predicted Number of Patients in Practices *Notes*. Log percentage difference is $\ln\left[{\hat{n}}_{j}\left(q_{06/7}\right)/{\hat{n}}_{j}\left(0\right)\right]=\ln\left(1+\delta_{j}\right)$ where ${\hat{n}}_{j}\left(q_{06/07}\right)$ is the number of patients predicted by the model in Table 2 to choose practice *j* when practices have their actual 2006/7 total QOF points and ${\hat{n}}_{j}\left(0\right)$ is the number predicted from a model with the same covariates and distance specification as Table 2 but with all practice qualities set to the same value. *δ*_*j*_ is the proportionate difference.

Finally, we illustrate the importance of quality in determining practice lists by using our baseline model to estimate the number of patients a practice would have if patients had no information about quality (or equivalently did not care about quality).^31^ We compare this to the predicted number of patients each practice has when patients have information about quality, using the estimates from the baseline model and the actual level of clinical quality used in estimation of the baseline model. The mean absolute change in estimated lists is 6%, so is not trivial. Figure 5, top panel, shows the distribution of the percentage change in the number of patients when quality is known across all practices compared to when it is not known. This is skewed to the left, with practices in the lower tail of the distribution predicted to lose a significant proportion of patients when quality is revealed. The lower part of Figure 5 plots the absolute gain against the 2006/7 QOF points of each practice. This clearly shows that practices which lose patients are those with low quality and those that gain are those with high quality. Our predictions therefore support the idea that the introduction of greater choice, or better information about quality, would lead to patients choosing higher quality practices.

## 6 Conclusion

The issue of whether choice and competition will increase the quality of health care services is both current and important. A prerequisite for increased competition to increase quality is that demanders are responsive to quality. We test whether this condition is satisfied in an important setting: the choice by patients of their family physicians. In the context we examine, these physician practices are important as they determine access to both primary and elective hospital health care services at zero direct monetary cost for the patient. Further, this context is not atypical: family doctors are important in many health care systems, they are the first (and often mandated) point of contact with health services and have low or zero direct user cost.

We examine the choices of 3.4 million individuals from amongst nearly 1,000 family doctor practices in a region of England. We find that clinical quality is important: individuals are more likely to choose practices with higher measured and published quality. They trade‐off quality against distance. The results are robust to alternative estimation methods, to the way in which distance is assumed to affect choice of practice and to possible restrictions on choice sets through the imposition of catchment areas and practices closing their lists for short periods to new patients.

We find some evidence of observed patient heterogeneity, with indication that low users (young men) care less about quality than other groups. We also find that those who live in small areas which are more deprived, or have less healthy or less educated populations, care less about quality relative to distance than others. But the size of the differences in the trade‐offs between quality and distance between these and other groups is relatively small. There was also little difference between those in areas with low and high proportions of the population of Asian origin in willingness to trade‐off quality for distance. This is in contrast to findings for some other services. Schneider *et al*. (1998, 2000) and Jacob and Lefgren (2007) report substantial race, class and income differences in household preferences for schools in the USA. In England, Burgess *et al*. (2014) find that higher income parents are more likely to value educational attainment whilst lower income parents are more likely to value proximity. Banks *et al*. (2010) and Smith *et al*. (2010), for the UK and the US respectively show strong correlations between an individual's numerical ability and their wealth, financial knowledge and their asset portfolio choices.

We exploit our estimates to look at the effect of quality on practice choice in order to provide an assessment of the impact of a policy to promote greater choice of family doctor. While the estimated effect of quality on the probability of any individual choosing a family practice is small, what matters for practice incentives is how many additional patients will be attracted by an increase in quality. This depends both on the effect of quality on the probability of choice by an individual and on the (large) number of individuals who could potentially choose the practice. Using the most conservative of our model specifications, we estimate that a 1 SD increase in measured clinical quality would, in the long run and with no response by rivals, attract approximately 17% more patients to a family practice. Even in the short run this implies large financial rewards as around 75% of practice income is linked to the number of individuals who register with the practice. We also show that, relative to a position with no quality information, practices that gain from the publication of quality are those with higher clinical quality. Finally, our estimated cross‐elasticities suggest that these primary care markets are very local: competition for practices comes from a small number of rivals located within a short distance.

Our findings support the argument that the promotion of greater choice of family doctor and provision of information about quality have the potential to benefit patients by increasing quality. Provided the marginal revenue from an additional patient sufficiently exceeds marginal cost, greater competition in this market means that family doctors have an incentive to improve their quality to attract patients. The similarity between patient groups in terms of trade‐offs between clinical quality and distance indicates that such a policy would benefit individuals across all age, gender, morbidity and socio‐economic status groups but would particularly benefit those who currently use practices of low clinical quality.

## Supporting information


**Data S1.**

## Notes

1 Our measures of practice quality are discussed in detail in subsection 2.3.

2 There is a related literature on provider competition in health care. The theoretical literature is generally supportive of the proposition that greater competition improves quality if prices are regulated (Gaynor, 2006), though there are caveats about the role of imperfect information and the required assumptions about provider cost functions and patient preferences (Gravelle, 1999; Gravelle and Masiero, 2000; Karlsson, 2007; Brekke *et al*., 2010; Gravelle *et al*., 2012; Halonen and Propper, 2012). Most empirical studies find that when providers face fixed prices, greater competition is associated with higher quality (for reviews, see Gaynor and Town, 2012; Gravelle *et al*., 2012). For England, Cooper *et al*. (2011) and Gaynor *et al*. (2013) report that the lifting of restrictions on patient choice of hospital led to faster reductions in acute myocardial infarction (AMI) mortality for providers facing more competition. In an exploratory cross‐sectional study of competition in primary care, Pike (2010) found that practices with more rivals within 500 m have higher quality (as measured by patient satisfaction and a measure of clinical care).

3 In the absence of any measures of clinical quality such attributes may be used by consumers as signals of a better match and so higher clinical quality.

4 In 2009/10 there were 26,420 GP contractors (i.e. joint owners rather than salaried employees) in England with average gross income £287,1001, expenses of £168,700 and net income of £109,400. There were 2,066 registered patients per GP contractor. See: GP Earnings and Expenses 2009/10, http://www.hscic.gov.uk/pubs/gpearnex0910 (last accessed: 10 March 2015); General and Personal Medical Services, England 2001–11, http://www.hscic.gov.uk/catalogue/PUB05214 (last accessed: 10 March 2015).

5 House of Commons, Hansard Written Answers for 28 April 2008.

6 GPs can deregister patients if there is a fundamental breakdown in their relationship. It has been estimated that each year 0.1% of patients are deregistered (Munro *et al*., 2002). If a patient cannot find a practice prepared to accept them, they can ask their PCT to find them a practice and PCTs can assign patients to practices. Around 0.5% of patients are assigned to practices (Audit Commission, 2004).

7
http://www.nhs.uk/choiceintheNHS/Yourchoices/GPchoice/Pages/ChoosingaGP.aspx. Detailed information on performance of practices in an area under the national P4P scheme is also available via http://www.qof.ic.nhs.uk/search/ and information from surveys of patient satisfaction is available at http://www.gp-patient.co.uk/info/ (all last accessed 10 March 2015).

8 The NHS Choices website notes: ‘The idea is to provide you with greater choice and to improve the quality of GP services over time, as GPs providing a good service are naturally more popular’. http://www.nhs.uk/NHSEngland/AboutNHSservices/doctors/Pages/patient-choice-GP-practices.aspx (accessed 27 November 2014).

9 On average over England the population registered with general practices is about 7% greater than estimates of the population derived from the decennial population census (Ashworth *et al*., 2005). The difference is due to lags in the updating of patient registration data when patients die or change practice. Since general practices are paid according to their registered lists it is appropriate to model the determinants of the number of patients registered with practices as we wish to examine whether practices are paid more, via larger lists, when their quality is greater.

10 The ADS includes patients resident in England and registered with practices located in Wales and Scotland.

11 There is no reliable information about individuals who are not registered with NHS general practices but they are likely to be a very small proportion of patients. For example, Davies *et al*. (2013) estimate that 0.5% of hospital admissions are of patients not registered with a general practice. Our model provides consistent estimates of the effect of quality on choices amongst NHS practices for individuals (the vast majority) who choose to register with an NHS practice.

12 Data on the type of contract are missing for 13 practices and rather than reduce the number of practices we assume they had GMS contracts and include a dummy variable indicating that the contract status is imputed.

13 Appendix A discusses other measures of quality and the results from a set of robustness checks using these measures. The measures include sub‐domains of the QOF, different years of the QOF, indices derived from the raw QOF data, practice emergency hospital admission rates for conditions which should be treatable in primary care and responses from patient satisfaction surveys.

14 The domains, points and indicators in 2006/7 were as follows. Clinical (80 indicators covering 19 conditions, carrying 655 points in total), organisation (43 indicators carrying 181 points for record keeping, medicines management, education and training,), patient experience (four indicators carrying 108 points for length of consultations and having undertaken patient surveys) and additional services (eight indicators carrying 36 points for services including cervical screening, child health surveillance, maternity and contraception). The holistic care indicator carried up to 20 points on the basis of performance in the third worst condition in the clinical domain. The data to compute these indicators are extracted directly from the patients’ electronic health records held by each practice.

15 Since the choice set for patients in LSOAs is defined only by distance, it satisfies the uniform conditioning property that it is not affected by which practices are chosen by patients in that LSOA. McFadden (1978) shows uniform conditioning is sufficient for consistency in the conditional logit model.

16 There were 981 practices in East Midlands choice sets. We drop eight practices without 2006/7 QOF data.

17 We do not have data on ethnicity or first language of GPs but the majority of doctors trained outside Europe will not have English as a first language.

18 Since utility depends on a cubic function of distance, the marginal rate of substitution varies across practices and LSOAs: $MRS_{qd}^{aj}=-\left(\partial u_{iaj}/\partial d_{aj}^{}\right)/\left(\partial u_{iaj}/\partial q_{j}^{}\right)=-{\hat{\beta }}_{q}/\left({\hat{\beta }}_{d}+2{\hat{\beta }}_{d^{2}}d_{aj}+3{\hat{\beta }}_{d^{3}}d_{aj}^{2}\right)$. We report the *MRS*_*qd*_ computed at the mean distance to the practices from LSOA centroids. Following Hole (2007), we estimate standard errors using the delta method.

19 As PCTs boundaries may reflect physical features that are hard to cross, we interpret the coefficient on PCT as a (non‐linear) distance parameter.

20 This literature suggests that female patients prefer consultations with female GPs, so we should, and do, find individuals are more likely to choose practices with a higher proportion of female GPs. GPs who have qualified outside Europe are less likely to have English as a first language and so may be perceived as being less able to communicate effectively with patients. The positive effect of a practice opting‐out of providing services to patients outside normal working hours may seem paradoxical. However, patients at a practice which has opted‐out will not necessarily experience worse access. When practices opt‐out the responsibility for providing out‐of‐hours care for their patients passes to the PCT. This need not lead to a reduction in the availability of out‐of‐hours care for patients compared to practices which do not opt‐out because much of the out‐of‐hours care is subcontracted to deputising services. It may also be that practices which opt‐out are then able to provide better care during normal hours. Individuals may prefer practices with PMS contracts because PMS contracts usually require the practice to provide additional services.

21 A model using the log of distance had a slightly smaller coefficient on quality (0.00216***) but worse overall fit (McFadden R^2^ = 0.3655). Results for all models are available in Appendix Table C8.

22 Alternatively, it may be the case that new residents are richer, healthier or better educated – all factors which the results in Table 4 suggest increase the valuation of quality relative to distance.

23 We also estimated models in which the choice set for an LSOA was defined as the set of all practices within 10 km and which had at least a specified numbers of patients from the LSOA on its list. This ensured that the LSOA was within the practice catchment area at some point over the previous years. Although these choice sets are partly choice based and so do not satisfy the uniform conditioning property, the results were very similar to those from the baseline model (Santos *et al*., 2013).

24 Because our preferred distance specification is cubic the marginal disutility of distance (and the MRS) depend on LSOA to practice distance as well as patient preferences. The average distance to practices in the choice set varies across our models and to ensure that differences in the marginal disutility and in the MRS reflect differences in preferences rather than differences in distance, we use the mean LSOA to practice distances from the 10 km model to compute the marginal disutility of distance and the MRS for all models.

25 91.9% of residents in rural LSOAs choose a practice within 7 km and 91.5% of those in urban areas choose a practice within 3 km.

26 One corollary that proposed policy changes (as of 2015) to remove a practice's right to set catchment areas will not have a large effect.

27 We draw 100 random bootstrap samples of 2,870 LSOAs with replacement. For each bootstrap sample, we estimate the first‐stage quality model for the practices in the choice sets of LSOAs in the sample. We estimate the second stage choice model for each bootstrap sample of LSOAs, adding the residuals from the first stage quality regression. We then compute the SD of the 100 estimates of the second stage coefficients.

28 We also considered local input prices for labour, land and buildings, as measured by the Market Forces Factors (MFF) constructed by the Department of Health, as potential instruments. They were weak instru‐ments (the largest first stage F‐statistic, on the staff MFF, was 3.88) and had the wrong (positive) sign in the first stage, suggesting that there were unobserved area amenity factors which drive up input prices and attract a population for whom it is easier to achieve QOF points. Some of the other practices in the same PCT as the practice will be in the same LSOA choice sets. Their quality may affect demand for the practice. Using the mean quality of all other practices, both rivals and non‐rivals, in the PCT will mitigate this violation of the exclusion restriction. To examine this further, we estimated a 2SRI model using the average quality of non‐rival practices in the same PCT as the instrument. It was a weaker instrument than the average quality of all other practices in the PCT (first stage F = 2.22) but the second stage coefficient on instrumented quality was highly significant and nearly five times (0.0108, SE = 0.003) as large as the un‐instrumented quality coefficient. See Appendix Table C12.

29 Note that columns (5) and (6) are computed for practices which draw at least 99% of their patients from LSOAs in the East Midlands whereas columns (1)–(4) are based on all patients in East Midlands LSOAs. See Appendix B for the details of the computations.

30 Our estimates do not take account of the very small proportion (Davies *et al*., 2013) of the population who are not registered with a practice. Higher quality may induce some of them to register so these are likely to be slight underestimates.

31 This predicted list size is computed using coefficients from the baseline model with the quality coefficient set to zero. This is equivalent to setting all qualities to the same value (including zero).

## Appendix A Alternative Quality Measures

### A.1

#### A.1. Alternative Measures

Our baseline quality measure is total QOF points for 2006/7. We also consider the 2006/7 points obtained in the different domains of the QOF, and total QOF points for 2009/10 and the average of total QOF points for the four years 2006/7–2009/10.

The indicators in the QOF were based on evidence on the effects of the incentivised activities on patient health.^32^ But as a measure of clinical quality QOF points have two potential drawbacks. Up to 665 of the 1,000 QOF points are awarded for having disease registers and for the percentage of eligible patients in a disease area for whom various indicators are achieved.^33^ No points were awarded for achievement less than 40% and points increased linearly with percentage achievement above 40% up to an upper threshold ranging from 60% to 90%, with no points earned for further increases in achievement. In addition, some practices appear to have designated patients as ‘exceptions’ to increase their reported achievement (Gravelle *et al*., 2010). Thus, points are an imperfect measure of actual achievement on a clinical indicator. In robustness tests, we use the raw QOF data on clinical indicators to construct measures (reported achievement and population achievement) which are not affected by upper and lower thresholds and exception reporting.

We also examine non‐QOF‐based measures of quality. The first is a measure of the quality of practice disease management: the practice's total annual emergency admission rate for ambulatory care sensitive conditions (ACSCs), listed in Table C2. ACSCs are conditions for which good quality management in general practice should prevent emergency admissions for complications (Agency for Healthcare Quality and Research, 2007; Purdy *et al*., 2009). ACSCs admission rates are used as measures of access to good quality primary care in many healthcare systems.

The second set of non‐QOF measures are derived from the 2009 GP Patient Survey which was sent to a random sample of patients in all practices in England. We use the answers to three questions. The first question concerns general satisfaction (‘In general, how satisfied are you with the care you get at your GP surgery or health centre?’). The second question is about satisfaction with opening hours (‘How satisfied are you with the hours that your GP surgery or health centre is open?’). Patients answer both questions on a 5‐point scale and we use the proportion of the practice respondents who say they were ‘Very satisfied’ or ‘Fairly satisfied’. The third question asks patients ‘Would you recommend your GP surgery or health centre to someone who has just moved to your local area?’ and we use the proportion of respondents who report ‘Yes, would definitely recommend’ or ‘Yes, might recommend’, as opposed to ‘Not sure’, ‘No, would probably not recommend’, ‘No, would definitely not recommend’ or ‘Don't know’. These measures are based on an achieved sample of 5% of patients, whilst the ACSC admission rate and QOF points are based on all relevant patients. Descriptive statistics for the different quality measures are reported in Table C3.

There are reasonably high correlations amongst the QOF points measures (Table C4).^34^ Although the QOF was intended to improve care for long‐term conditions and to reduce hospital admissions, there is only a weak negative correlation between ACSCs and QOF points. This may be because there are both negative and positive correlations between admissions for particular ACSCs and the QOF clinical indicators for management of those conditions (Downing *et al*., 2007; Bottle *et al*., 2008; Dusheiko *et al*., 2011; Purdy *et al*., 2011). The three patient‐reported measures are reasonably highly correlated with each other but much less well correlated with the QOF measures. Finally, the reported achievement 2009/10 and population achievement 2009/10 measures, which use more of the information used to compute QOF clinical indicators, are highly though not perfectly correlated with total 2009/10 QOF points and with each other.

#### A.2. Choice Models with Alternative Quality Measures

We estimated practice choice models with these alternative quality measures with models including the same covariates and cubic distance specification as our baseline model. They had very similar coefficients for distance and other practice characteristics as our baseline model.

We first estimate a model using the points from the separate domains of the 2006/7 QOF (clinical, organisational, patient experience, additional services, holistic care). This gives a slightly better overall fit than models which use only a single QOF points measure (Table C5). However, two of the domains (holistic care and patient experience) have negative, though insignificant, effects (possibly because of collinearity amongst the five components). A model with only 2006/7 QOF clinical points had markedly smaller MRS against distance (0.0044; SE 0.0004) and slightly worse fit than the baseline model.

Second, we examine the choice of period for which the QOF points are reported. The QOF was introduced 2004/5 but the number of disease areas covered was increased from 10 to 19 in 2006/7, so that we think that measures based on performance from 2006/7 onwards provide a better measure of quality. If there were no switching costs then all patients on a practice list can be considered to have chosen it recently, given their information on the current characteristics of practices in their choice set. In this case, we would expect that current quality (2009/10 QOF points) would be a better predictor of choices than 2006/7 QOF points or 2006/7–2009/10 average QOF points. However, we find (Table C5) that replacing 2006/7 total QOF points with 2009/10 total QOF points reduces that model fit slightly compared to these models and the marginal rate of substitution between quality and distance is less precisely estimated (MRS 0.009; SE 0.0002). At the other extreme, if switching costs were so high that no patient switched practices unless they changed their LSOA of residence, then the observed practice lists would reflect decisions made by annual cohorts of new residents whose choices were dependent on quality observed in the year they arrived in the LSOA. The mean population inflow in the East Midlands is around 5% per annum suggesting that the average patient observed on 1 April 2010 chose their practice about 10 years previously. This implies that 2006/7 QOF points should be a better predictor of choice than average 2006/7–209/10 QOF points. We find that the model with average 2006/7–2009/10 QOF points has larger MRS of distance for quality (0.0044; SE 0.0004) but fits slightly less well than the baseline model with 2006/7 QOF points.

We constructed two further measures (reported and population achievement) from the raw QOF data. They allow for lower and upper thresholds and exception reporting but neither measure was significant when it replaced total 2006/7 QOF points in the baseline specification and reported achievement had a negative sign (Table C6). We also estimated models with the practice rate of emergency admissions for ambulatory sensitive conditions as a measure of quality. When it was the only explanatory it had the appropriate (negative) sign and was statistically significant. However, its coefficient was small and insignificant in the baseline specification with or without total QOF 2006/7 points.

In Table C7, we examine patient satisfaction with their practice as a measure of quality. When overall patient satisfaction is the only measure of quality and no other covariates included in the model, patient satisfaction is strongly correlated with choice of practice. But when we also include other practice characteristics and our baseline measure of clinical quality (total QOF 2006/7 points), the coefficient on overall patient satisfaction becomes negative and insignificant. Thus, patient satisfaction seems to summarise the effect of practice characteristics on patient utility, as suggested in Robertson *et al*. (2008) but makes no independent contribution to predicting patient choice of practice. The coefficient for patient satisfaction with access was statistically significant but of the wrong sign (negative) when entered as the only explanatory variable in the choice model. This may be because practices with high demand have longer waits for consultations. As with the overall satisfaction measure, satisfaction with access was insignificant when other practice characteristics and total QOF 2006/7 points were included in the model. The patient survey measure ‘would recommend’ is positively associated with choice but its coefficient fell six fold reduction when practice characteristics and total QOF 2006/7 points were added to the model.

## Appendix B Calculation of Effects

### B.1

#### B.1. Patients Gained

We report the estimated average number of additional patients a practice would receive from a 1 SD increase in practice characteristic *x*_*kj*_. We compute this by first computing the estimated average number gained from a one unit increase in the characteristic:

(B1) $$\frac{1}{n^{J*}}\sum_{j\in S^{J*}}^{}\left(\frac{\partial {\hat{n}}_{j}}{\partial x_{kj}}\right)=\frac{1}{n^{J*}}\sum_{j\in S^{J*}}^{}\left[\frac{\partial \left(\sum_{a\in S_{j}}n_{a}{\hat{P}}_{aj}\right)}{\partial x_{kj}}\right]=\frac{1}{n^{J*}}\sum_{j\in S^{J*}}^{}\left[\sum_{a\in S_{j}}^{}n_{a}{\hat{\beta }}_{k}{\hat{P}}_{aj}\left(1-{\hat{P}}_{aj}\right)\right],$$

where ${\hat{P}}_{aj}$ is the probability of patients from LSOA *a* choosing practice *j*, ${\hat{\beta }}_{k}$ is the estimated coefficient on characteristic *k*,* n*_*a*_ is the number of patients in LSOA *a* and ${\hat{n}}_{j}=\sum_{a\in S_{j}}^{}n_{a}{\hat{P}}_{aj}$ is the predicted number of patients choosing practice *j*. $S_{j}=\left\{a\left|j\in C_{a}\right.\right\}$ is the set of East Midlands LSOAs whose choice sets include practice *j*. *S* ^*J**^ is the set of *n* ^*J**^ (= 415) practices which draw at least 99% of their list from East Midland LSOAs.

About half the practices in the choice sets of the East Midlands LSOAs draw some of their patients from LSOAs outside the East Midlands. Since we do not estimate ${\hat{P}}_{aj}$ for these LSOAs, we compute the numbers gained for the 415 practices which draw at least 99% of their patients from the East Midlands LSOAs.

#### B.2. Cross‐practice Quality Elasticity and Distance

The estimated change in the number of patients choosing practice *j* if the quality of practice *m* increases is

(B2) $$\frac{\partial {\hat{n}}_{j}}{\partial q_{m}}=\frac{\partial \left(\sum_{a\in S_{j}\cap S_{m}}n_{a}{\hat{P}}_{aj}\right)}{\partial q_{m}}=-\sum_{a\in S_{j}\cap S_{m}}^{}n_{a}{\hat{\beta }}_{q}{\hat{P}}_{aj}{\hat{P}}_{am},$$

where ${\hat{P}}_{aj}$ is the probability of patients from LSOA *a* choosing practice *j,* ${\hat{P}}_{am}$ is the probability of patients from LSOA *a* choosing practice *m*,* n*_*a*_ is the population of LSOA *a*. $S_{j}=\left\{a\left|j\in C_{a}\right.\right\}$, $S_{m}=\left\{a\left|m\in C_{a}\right.\right\}$ are the sets of East Midlands LSOAs whose choice sets include practice *j*,* m*. The quality of practice *m* affects demand for practice *j* only by changing demand for *j* from LSOAs which have choice sets which include both *j* and *m*. ${\hat{\beta }}_{q}$ is the estimated coefficient on quality.

The cross‐elasticity of demand for practice *j* with respect to the quality of practice *m* is

(B3) $$\varepsilon_{jm}^{q}=\frac{\partial {\hat{n}}_{j}}{\partial q_{m}}\frac{q_{m}}{{\hat{n}}_{j}}=\frac{\partial \left(\sum_{a\in S_{j}\cap S_{m}}n_{a}{\hat{P}}_{aj}\right)}{\partial q_{m}}\frac{q_{m}}{{\hat{n}}_{j}}=-\sum_{a\in S_{j}\cap S_{m}}^{}n_{a}{\hat{\beta }}_{q}{\hat{P}}_{aj}{\hat{P}}_{am}\frac{q_{m}}{{\hat{n}}_{j}}=-\frac{{\hat{\beta }}_{q}q_{m}}{{\hat{n}}_{j}}\sum_{a\in S_{j}\cap S_{m}}^{}n_{a}{\hat{P}}_{aj}{\hat{P}}_{am}.$$

We compute $\varepsilon_{jm}^{q}$ where *j* is in the set of practices *S* ^*J**^ which draw at least 99% of their list from East Midland LSOAs (as in computation for the patient gain on own quality) and *m* is each of the other practices included in at least one choice set of the set of practices in *S* ^*J**^. There are 973 practices in the data set for our preferred specification but we compute fewer than 492 × 972 values of $\varepsilon_{jm}^{q}$ as *S*_*j*_ ∩ *S*_*m*_ will be empty if the distance *d*_*jm*_ between practice *j* and *m* is greater than >20 km since LSOA choice sets have radius of at most 10 km.

We plot $\varepsilon_{jm}^{q}$ against the distance *d*_*jm*_ between practice *j* and practice *m* (Figure 4). We expect $\varepsilon_{jm}^{q}$ to be smaller in absolute value as *d*_*jm*_ is greater since as *d*_*jm*_ increases the set of LSOAs in *S*_*j*_ ∩ *S*_*m*_ will become smaller and so the increase in *q*_*m*_ will affect fewer LSOAs which include practice *j* in their choice sets. This respects Tobler's first law of geography ‘Everything is related to everything else, but near things are more related than distance things’.

#### B.3. Average Marginal Effect

We report (Table 8) the average change in probability of patients from an LSOA choosing a practice from a unit increase in practice characteristic *x*_*kj,*_ that is the average marginal effect.

The marginal effect for each of the *n*_*a*_ individuals in LSOA is

(B4) $$ME_{ajk}={\hat{\beta }}_{k}{\hat{P}}_{aj}\left(1-{\hat{P}}_{aj}\right),$$

where ${\hat{P}}_{aj}$ is the probability of patients from LSOA *a* choosing practice *j* and ${\hat{\beta }}_{k}$ is the estimated coefficient on characteristic *k*.

The average of *ME*_*ajk*_ over all the patients is

(B5) $$AME_{k}=\frac{\sum_{a}^{N^{A}}\sum_{j}^{N^{J}}ME_{ajk}n_{a}}{\sum_{a}^{N^{A}}n_{a}N^{Ca}}=\frac{\sum_{a}^{N^{A}}n_{a}\sum_{j}^{N^{J}}ME_{ajk}}{\sum_{a}^{N^{A}}n_{a}N^{Ca}}=\sum_{a}^{N^{A}}\left(\frac{n_{a}}{\sum_{a}^{N^{A}}n_{a}N^{Ca}}\right)\sum_{j}^{N^{J}}ME_{ajk},$$

where *n*_*a*_ is the number of patients in LSOA *a, C*_*a*_ is the choice set of LSOA *a*,* N* ^*Ca*^ is the number practices in *C*_*a*_.

We compute the numbers gained for the 415 practices which draw at least 99% of their patients from the East Midlands LSOAs.

## Appendix C Additional Tables: Data, Alternative Quality Models, Robustness Checks

### C.1

**Table C1:** Data Sources

Data set	Variables	Source
Attribution Data Set	Patients in each LSOA by age/gender on each practice list	NHS England*
Quality and Outcomes Framework	QOF points total and by indicator; numbers for whom indicator achieved, exceptions	www.ic.nhs.uk/statistics-and-data-collections/audits-and-performance/the-quality-and-outcomes-framework
GP Patient Survey	Patient satisfaction with practice	www.gp-patient.co.uk/archive_weighted/practicereport
Hospital Episode Statistics	Emergency admissions for ambulatory care sensitive conditions	www.hesonline.nhs.uk * Health and Social Care Information Centre*
General Medical Service Statistics	Age, gender, country of qualification of GPs, practice contract, out of hour status, dispensing status, location	Health and Social Care Information Centre*
NHS Choices	Location of branch practices	www.nhs.uk/Pages/HomePage.aspx
Neighbourhood Statistics	Socio‐economic and demographic measures at LSOA level. Population turnover at MSOA level	www.neighbourhood.statistics.gov.uk/dissemination
Index of Multiple Deprivation	LSOA income deprivation, proportion adults no educational qualification	www.communities.gov.uk/publications/communities/indicesdeprivation07
Office of National Statistics	LSOA rurality classification	www.ons.gov.uk/ons/guide-method/geography/products/area-classifications/index.html

Notes

*Data released under Data Sharing Agreement requiring that it is not shared with a third party. HES Copyright © 2006–2010, reused with the permission of The Health and Social Care Information Centre. All rights reserved.

**Table C2:** Ambulatory Care Sensitive Conditions: ICD10 Codes

Condition	ICD10 codes
Asthma	J45 J46
Circulatory system	I110 I130 I132 I10 I119 I129 I139
COPD	J20 J41 J42 J43 J44 J47 J40
Stroke/LVD	I60 I61 I63 I64 I66 I672 I698 R470
EPILEPSY	G40 G41 R56 G253 R568
CHD/LVD	I20 I240 I248 I249 I25 R072 I21 I22 I110 I130 I132 I255 I50 J81
Diabetes	E110 E111 E112 E113 E114 E115 E116 E117 E118 E119 E10 E120 E121 E122 E128 E130 E131 E132 E133 E134 E135 E136 E137 E138 E140 E141 E142 E143 E144 E145 E146 E147 E148
DKD or Dementia	N03 F00 F01 F02 F03
Alcohol‐related disease	F10
Perforated appendix	K350 K351
Dehydration & gastroenteritis	A020 A04 A059 A072 A080 A081 A083 A082 A084 A085 A09 E86 K520 K521 K522 K528 K529
Cellulitis	I891 L010 L011 L020 L021 L022 L023 L024 L028 L029 L03 L04 L080 L088 L089 L88 L980
ENT	H66 H67 J02 J03 J040 J06 J312
Gangrene	R02
Influenza and pneumonia	A481 A70 J10 J11 J120 J121 J122 J128 J129 J13 J14 J153 J154 J157 J159
	J160 J168 J18 J181 J189 J180 J188
Iron‐deficiency anaemia	D460 D461 D463 D464 D501 D508 D509 D510 D511 D512 D513 D518 D520 D521 D528 D529 D531 D571 D580 D581 D590 D591 D592 D599 D601 D608 D609 D610 D611 D640 D641 D642 D643 D644 D648 E40 E41 E42 E43 E550 E643
Other vaccine preventable diseases	A35 A36 A37 A80 B05 B06 B161 B169 B180 B181 B26 G000 M014
Pelvic inflammatory	N70 N73 N74
Perforated/bleeding ulcer	K20 K210 K219 K221 K226 K250 K251 K252 K254 K255 K256 K260 K261 K262 K264 K265 K266 K270 K271 K272 K274 K275 K276 K280 K281 K282 K284 K285 K286 K920 K921 K922
Atrial fibrillation and flutter	I498 R000 I471 I479 I499 R002 R008 I495
Constipation	K590
Urinary infection	N11 N136 N10 N151 N159 N12 N390 N300 N309 N308
Fracture proximal femur	S722 S720 S721
Peripheral vascular disease	I73 I738 I739
Failure to thrive	R629
Dyspepsia	K21 K30
Hypokalemia	E876
Low birth weight	P050 P052 P059 P072 P073
Migraine	G43 G440 G441 G443 G444 G448 R51
Tuberculosis	A15 A16 A17 A18 A19
Dental conditions	A690 K098 K099 K02 K03 K04 K05 K06 K08 K12 K13

**Table C3:** Descriptive Statistics for Quality Measures

	Mean	Median	SD	Minimum	Maximum	*N*
*Quality measures*						
QOF 2006/7 total points	955.5	980.1	64.6	426.5	1,000.0	973
QOF 2006/7 clinical points	632.6	646.4	36.9	330.5	655.0	973
QOF 2006/7 organisational points	166.3	174.2	21.2	13.2	181.0	973
QOF 2006/7 patient experience points	103.1	108.0	16.4	0.0	108.0	973
QOF 2006/7 additional services points	35.2	36.0	2.9	6.0	36.0	973
QOF 2006/7 holistic care points	18.3	19.7	3.1	0.0	20.0	973
QOF 2009/10 total points	940.3	947.0	47.5	545.5	1,000	981
Average QOF total points 2006/7–2009/10	954.7	966.4	45.2	545.5	1,000	971
Population achievement 2009/10	0.720	0.724	0.044	0.379	0.829	981
Reported achievement 2009/10	0.782	0.790	0.043	0.484	0.887	981
ACSCs 2009/10 per 10,000	259	250	76	28	628	966
Overall patient satisfaction 2009	0.893	0.904	0.062	0.572	0.992	981
Satisfaction with opening hours 2009	0.798	0.801	0.065	0.454	0.970	981
Prop patients would recommend practice 2009	0.823	0.843	0.105	0.383	0.992	981

**Table C4:** Correlations Between Quality Measures

	QOF 2006/7 organisational	QOF 2006/7 patient experience	QOF 2006/7 additional services	QOF 2006/7 holistic	QOF 2006/7 total points	QOF 2009/10 total points	Average QOF total points 2006/7–2009/10	Population achievement 2009/10	ACSCs 2006/7	ACSCs 2009/10	Proportion patients who would recommend practice 2009	Overall patient satisfaction 2009	Satisfaction with opening hours 2009
QOF 2006/7 organisational	0.5281	1											
QOF 2006/7 patient experience	0.3502	0.4562	1										
QOF 2006/7 additional services	0.3864	0.5272	0.5851	1									
QOF 2006/7 holistic	0.9187	0.4941	0.313	0.3486	1								
QOF 2006/7 total points	0.894	0.7923	0.644	0.6033	0.8292	1							
QOF 2009/10 total points	0.5226	0.3486	0.2028	0.2428	0.5004	0.4988	1						
Average QOF total points 2006/7–2009/10	0.8169	0.6525	0.4551	0.4643	0.7745	0.855	0.8319	1					
Population achievement 2009/10	0.4798	0.239	0.1185	0.1818	0.458	0.4123	0.7814	0.6559	1				
ACSCs 2006/7	−0.0892	−0.0611*	−0.083	−0.082	−0.0703	−0.099	−0.092	−0.1024	−0.0491*	1			
ACSCs 2009/10	−0.0991	−0.0651	−0.0355*	−0.0732	−0.0839	−0.0943	−0.1461	−0.1356	−0.0863	0.7183	1		
Proportion patients who would recommend practice 2009	0.1573	0.1506	0.1707	0.1479	0.1823	0.1978	0.3354	0.3047	0.1484	−0.1395	−0.1494	1	
Overall patient satisfaction 2009	0.0379*	0.0644	0.0338*	0.0293*	0.0417*	0.0546*	0.2347	0.1318	0.1063	0.0277*	−0.0231*	0.6509	1
Satisfaction with opening hours 2009	0.2175	0.1848	0.2022	0.1778	0.2382	0.2554	0.3572	0.351	0.1626	−0.178	−0.1889	0.9135	0.5485

Notes

*Indicates correlation has p ≥ 0.05 that is correlation *not* statistically significant at 5%.

**Table C5:** Choice Models with Alternative QOF Points‐Based Quality Measures

	Co‐efficient	Co‐efficient	Co‐efficient	Co‐efficient	Co‐efficient
QOF 2006/7 total points	0.00222***				
	(0.00016)				
QOF 2009/10 total points		0.000690***			
		(0.00019)			
QOF av four years 2006/7–2009/10			0.00315***		
			(0.00023)		
QOF 2006/7 clinical points				0.00327***	0.00228***
				(0.00028)	(0.00059)
QOF 2006/7 organisational points					0.00268***
					(0.00047)
QOF 2006/7 patient experience points					−0.000342
					(0.00064)
QOF 2006/7 additional services points					0.0192***
					(0.00396)
QOF 2006/7 holistic care points					−0.00275
					(0.00617)
Distance	−0.7512***	−0.7523***	−0.7519***	−0.7507***	−0.7508***
	(0.0163)	(0.0162)	(0.0162)	(0.0163)	(0.0162)
BIC	11,597,725	11,687,958	11,664,682	11,607,006	11,594,414
McFadden R^2^	0.3991	0.3974	0.3985	0.3986	0.3993
*N* practices	973	981	971	973	973
*N* patients	3,350,561	3,358,351	3,358,351	3,350,561	3,350,561
MRS	0.0030***	0.0009***	0.0042***	0.0044***	
	(0.0002)	(0.0002)	(0.0003)	(0.0004)	

Notes

Conditional logit models of choice of practice. All models have the same covariates, distance specification and choice set definitions as in Table 2. Distance coefficient is ${\hat{\beta }}_{d}+2{\hat{\beta }}_{d^{2}}\overset{¯}{d}+3{\hat{\beta }}_{d^{3}}\bar{d^{2}}$ where $\overset{¯}{d}$ and $\bar{d^{2}}$ are computed as the mean of the LSOA centroid to practice distance and squared distance for the whole sample. MRS is the coefficient on quality divided by the distance coefficient. Number of LSOAs is 2,870 in all models. Standard errors clustered at LSOA level are in parentheses. *p < 0.05, **p < 0.01, ***p < 0.001.

**Table C6:** Choice Models with Other QOF Derived Quality Measures and With ACSC Emergency Admissions

	1*a*	1*b*	1*c*	2*a*	2*b*	2*c*	3*a*	3*b*	3*c*
QOF 2006/7 Total points			0.00256***			0.00252***			0.00226***
			(0.00017)			(0.00016)			(0.00016)
PA 2009/10	0.199	0.00399	−1.158***						
	(0.232)	(0.191)	(0.203)						
RA 2009/10				0.308	−0.167	−1.117***			
				(0.229)	0.189	(0.195)			
ACSCs 2006/7							−0.00097***	−0.00020	−0.00009
							(0.00016)	(0.00012)	(0.00013)
Distance		−0.7526***	−0.7512***		−0.7526***	−0.7514***		−0.7530***	−0.7524***
		(0.0162)	(0.0163)		(0.0162)	(0.0162)		(0.0163)	(0.0163)
BIC	19,395,420	11,693,489	11,596,321	19,395,151	11,693,399	11,596,570	19,303,987	11,627,379	11,559,388
McFadden R^2^	0.0000	0.3971	0.3992	0.0000	0.3971	0.3992	0.0006	0.3980	0.3995
*N* LSOAs	2,870	2,870	2,870	2,870	2,870	2,870	2,869	2,869	2,869
*N* practices	981	981	973	981	981	973	974	974	970
*N* patients	3,358,351	3,358,351	3,350,561	3,358,351	3,358,351	3,350,561	3,348,152	3,348,152	3,342,041

Notes

Conditional logit models of choice of practice. Models 1*a*, 2*a*, 3*a* only contain the stated quality measure. Models 1*b*, 1*c*, 2*b*, 2*c*, 3*b*, 3*c* have the same covariates, distance specification and choice set definitions as in Table 2. Distance coefficient is ${\hat{\beta }}_{d}+2{\hat{\beta }}_{d^{2}}\overset{¯}{d}+3{\hat{\beta }}_{d^{3}}\bar{d^{2}}$ where $\overset{¯}{d}$ and $\bar{d^{2}}$ are computed as the mean of the LSOA centroid to practice distance and squared distance for the whole sample. PA: population achievement is $\sum_{q}\left(N_{jq}/\left(D_{jq}+E_{jq}\right)\right)\left(\pi_{q}^{\max}\right)/\left(\sum_{q}\pi_{q}^{\max}\right)$ where *N*_*jq*_ is the number of patients for whom QOF clinical indicator *q* is achieved by practice *j*,* D*_*jq*_ is the number of patients who are declared eligible for indicator *q*,* E*_*jq*_ is the number of patients exception reported for indicator *q* by practice *j*. $\pi_{q}^{\max}$ is the maximum number of points achievable for indicator *q*. RA: reported achievement for practice *j* is $\sum_{q}\left(N_{jq}/D_{jq}\right)\left(\pi_{q}^{\max}\right)/\left(\sum_{q}\pi_{q}^{\max}\right)$ ACSCs is the number of emergency admissions for ambulatory care sensitive conditions of practice patients per 10,000 patients registered with the practice. Standard errors clustered at LSOA level are in parentheses. *p < 0.05, **p < 0.01, ***p < 0.001.

**Table C7:** Choice Models with Patient Reported Experience Quality Measures

	1*a*	1*b*	2*a*	2*b*	3*a*	3*b*
QOF 2006/7 total points		0.00224***		0.00224***		0.00212***
		(0.00016)		(0.00016)		(0.00016)
Overall satisfaction	2.017***	−0.146				
	(0.227)	(0.160)				
Access satisfaction			−0.821***	−0.216		
			(0.188)	(0.138)		
Would recommend					2.014***	0.337**
					(0.143)	(0.105)
Distance		−0.7511***		−0.7508***		−0.7512***
		(0.0163)		(0.0163)		(0.0163)
BIC	19,360,123	11,597,634	19,388,050	11,597,404	19,296,768	11,596,220
McFadden R^2^		0.3991		0.3991		0.3992

Notes

Conditional logit models of choice of practice. Models 1*a*, 2*a*, 3*a* only contain the stated patient experience measure. Models 1*b*, 2*b*, 3*b* also contain have the same covariates, distance specification and choice set definitions as in Table 2. Distance coefficient is ${\hat{\beta }}_{d}+2{\hat{\beta }}_{d^{2}}\overset{¯}{d}+3{\hat{\beta }}_{d^{3}}\bar{d^{2}}$ where $\overset{¯}{d}$ and $\bar{d^{2}}$ are computed as the mean of the LSOA centroid to practice distance and squared distance for the whole sample. Number of LSOAs is 2870, number of practices is 973, number of patients is 3,350,561 in all models. Standard errors clustered at LSOA level are in parentheses. *p < 0.05, **p < 0.01, ***p < 0.001.

**Table C8:** Choice Models with Alternative Distance Specifications

	Linear	Quadratic	Cubic	Quartic	Quintic	Log distance
QOF 2006/7 points	0.00231***	0.00221***	0.00222***	0.00222***	0.00222***	0.00216***
	(0.00016)	(0.00016)	(0.00016)	(0.00016)	(0.00016)	(0.00018)
Distance km	−0.963***	−1.424***	−1.606***	−1.430***	−1.410***	
	(0.01189)	(0.01804)	(0.04044)	(0.07144)	(0.12245)	
Distance squared		0.0683***	0.126***	0.0318	0.0166	
		(0.00226)	(0.01189)	(0.03442)	(0.08575)	
Distance cubed			−0.00447***	0.0125*	0.0171	
			(0.00091)	(0.00607)	(0.02466)	
Distance power four				−0.000955**	−−0.00154	
				(0.00035)	(0.00303)	
Distance power five					0.0000257	
					(0.00013)	
Ln distance						−1.851***
						(0.02036)
Distance	−0.9633***	−0.8161***	−0.7512***	−0.7406***	−0.7371***	−0.4163***
	(0.0119)	(0.0092)	(0.0163)	(0.0170)	(0.0249)	(0.0046)
MRS	0.0024***	0.0027***	0.0030***	0.0030***	0.0030***	0.0052***
	(0.0002)	(0.0002)	(0.0002)	(0.0002)	(0.0002)	(0.0004)
BIC	11,779,955	11,603,666	11,597,725	11,596,060	11,596,070	12,246,338
McFadden R^2^	0.3897	0.3988	0.3991	0.3992	0.3992	0.3655

Notes

Conditional logit models of choice of practice. All models have the same covariates as in Table 2. The distance coefficient is the derivative of the utility function evaluated using the estimated coefficients and the mean of the powers of LSOA to practice distance for the whole sample. Standard errors clustered at LSOA level are in parentheses. *p < 0.05, **p < 0.01, ***p < 0.001.

**Table C9:** Comparison of Practice Choice Models by Age and Gender Groups: Full Results

	All	Female all	F 24–34	F35–44	F 45–64	F 65–74	F 75plus
(*a*) *Female patients*							
QOF 2006/7 total points	0.00222***	0.00236***	0.00208***	0.00257***	0.00251***	0.00228***	0.00216***
	(0.00016)	(0.00016)	(0.00016)	(0.00017)	(0.00017)	(0.00019)	(0.00021)
Distance	−0.7512***	−0.7575***	−0.7708***	−0.7499***	−0.7433***	−0.7679***	−0.8182***
	(0.0163)	(0.0165)	(0.0175)	(0.0165)	(0.0169)	(0.0178)	(0.0198)
Practice in different PCT	−0.881***	−0.892***	−0.898***	−0.907***	−0.888***	−0.875***	−0.889***
	(0.04436)	(0.04491)	(0.04901)	(0.04546)	(0.04646)	(0.04859)	(0.05336)
Average GP Age (yrs)	−0.0257***	−0.0269***	−0.0263***	−0.0245***	−0.0269***	−0.0287***	−0.0301***
	(0.00163)	(0.00169)	(0.00174)	(0.00176)	(0.00176)	(0.00196)	(0.00206)
Prop Female GPs	0.239***	0.275***	0.360***	0.304***	0.259***	0.189***	0.230***
	(0.03364)	(0.03466)	(0.03919)	(0.03607)	(0.03572)	(0.04068)	(0.04358)
Prop Non‐European qualified GPs	−0.527***	−0.551***	−0.437***	−0.530***	−0.561***	−0.637***	−0.665***
	(0.02809)	(0.02910)	(0.02950)	(0.03062)	(0.03074)	(0.03441)	(0.03729)
Opted out of 24 hours obligation	0.160***	0.155***	0.166***	0.147***	0.173***	0.134**	0.118**
	(0.03748)	(0.03813)	(0.04160)	(0.04093)	(0.03974)	(0.04157)	(0.04335)
PMS contract	0.148***	0.142***	0.202***	0.151***	0.142***	0.100**	0.0864*
	(0.03317)	(0.03376)	(0.03662)	(0.03630)	(0.03534)	(0.03693)	(0.03800)
MRS	0.00295***	0.00312***	0.00270***	0.00342***	0.00338***	0.00297***	0.00264***
	(0.00022)	(0.00023)	(0.00022)	(0.00024)	(0.00025)	(0.00026)	(0.00026)
BIC	11,597,725	5,769,627	1,054,605	1,179,122	2,110,832	719,211	701,624
McFadden R^2^	0.3991	0.4059	0.4005	0.4040	0.3995	0.4107	0.4336
*N* patients	3,350,561	1,688,960	295,329	341,336	616,961	217,072	218,176

	Male all	M 25–34	M 35–44	M 45–64	M 64–74	M 75 plus
(*b*) *Male patients*						
QOF 2006/7 total points	0.00208***	0.00137***	0.00219***	0.00234***	0.00214***	0.00234***
	(0.00015)	(0.00018)	(0.00016)	(0.00017)	(0.00019)	(0.00020)
Distance	−0.7447***	−0.7550***	−0.7530***	−0.7396***	−0.7426***	−0.7843***
	(0.0161)	(0.0178)	(0.0162)	(0.0164)	(0.0176)	(0.0184)
Practice in different PCT	−0.871***	−0.851***	−0.873***	−0.886***	−0.859***	−0.845***
	(0.04426)	(0.06377)	(0.04522)	(0.04535)	(0.04875)	(0.04929)
Average GP age (years)	−0.0245***	−0.0240***	−0.0225***	−0.0241***	−0.0274***	−0.0287***
	(0.00159)	(0.00169)	(0.00166)	(0.00164)	(0.00194)	(0.00198)
Prop female GPs	0.202***	0.256***	0.218***	0.182***	0.153***	0.178***
	(0.03300)	(0.04010)	(0.03425)	(0.03388)	(0.04034)	(0.04183)
Prop Non‐Europ qualif GPs	−0.504***	−0.452***	−0.478***	−0.500***	−0.578***	−0.625***
	(0.02740)	(0.03071)	(0.02829)	(0.02868)	(0.03336)	(0.03508)
Opted out of 24 hours obligation	0.165***	0.231***	0.153***	0.157***	0.133**	0.123**
	(0.03714)	(0.04424)	(0.03886)	(0.03839)	(0.04168)	(0.04087)
PMS contract	0.153***	0.243***	0.166***	0.135***	0.0878*	0.0913*
	(0.03285)	(0.03865)	(0.03432)	(0.03405)	(0.03706)	(0.03595)
MRS	0.00279***	0.00181***	0.00291***	0.00316***	0.00289***	0.00298***
	(0.00021)	(0.00024)	(0.00022)	(0.00023)	(0.00027)	(0.00026)
BIC	5,827,061	1,144,601	1,270,483	2,215,871	696,124	495,759
McFadden R^2^	0.3924	0.3824	0.3933	0.3907	0.4001	0.4139
*N* patients	1,661,426	309,837	358,003	636,112	207,247	150,139

Notes

Conditional logit models of choice of practice. All models have the same covariates as in Table 2. The distance coefficient is the derivative of the utility function evaluated using the estimated coefficients and the mean of the powers of LSOA to practice distance for the whole sample. Standard errors clustered at LSOA level are in parentheses. *p < 0.05, **p < 0.01, ***p < 0.001.

**Table C10:** Choice Models for LSOA Samples Stratified by Socio‐economic Characteristics and Population Mobility: Full Results

	Urban	Rural	Lower inc depriv	Higher inc depriv	Better education	Worse education
QOF 2006/7 total points	0.00224***	0.00188***	0.00249***	0.00144***	0.00238***	0.00181***
	(0.00014)	(0.00046)	(0.00019)	(0.00026)	(0.00019)	(0.00028)
Distance	−0.8730***	−0.6706***	−0.7714***	−0.7755***	−0.7450***	−0.8383***
	(0.0172)	(0.0263)	(0.0175)	(0.0350)	(0.0179)	(0.0320)
Practice in different PCT	−0.865***	−0.842***	−0.862***	−0.988***	−0.863***	−0.941***
	(0.04602)	(0.09952)	(0.04694)	(0.13059)	(0.04887)	(0.10134)
Average GP age (year)	−0.0256***	−0.0308***	−0.0286***	−0.0177***	−0.0279***	−0.0165***
	(0.00168)	(0.00494)	(0.00196)	(0.00264)	(0.00191)	(0.00286)
Prop female GPs	0.260***	0.116	0.239***	0.228***	0.220***	0.299***
	(0.03347)	(0.11182)	(0.04103)	(0.05126)	(0.03883)	(0.06451)
Prop Non‐Europ qualif GPs	−0.503***	−0.660***	−0.619***	−0.345***	−0.590***	−0.360***
	(0.02814)	(0.10125)	(0.03426)	(0.04579)	(0.03273)	(0.05376)
Opted out	0.251***	−0.0678	0.122**	0.384***	0.110**	0.379***
	(0.03659)	(0.08787)	(0.04170)	(0.07258)	(0.04231)	(0.07176)
PMS contract	0.244***	−0.161*	0.0945*	0.419***	0.0968*	0.373***
	(0.03219)	(0.08160)	(0.03693)	(0.06409)	(0.03800)	(0.06042)
MRS	0.00256***	0.00281***	0.00323***	0.00186***	0.00320***	0.00216***
	(0.00017)	(0.00070)	(0.00026)	(0.00034)	(0.00026)	(0.00033)
BIC	9327639	2243349	8954600	2610534	9289002	2295492
McFadden R^2^	0.3699	0.5014	0.4138	0.3515	0.3997	0.4001
*N* LSOA	2,100	770	2,295	575	2,295	575
*N* GP practices	796	854	971	672	968	738
*N* patients	2,404,472	946,089	2,711,039	639,522	2,701,394	649,167

	Better health	Worse health	Less Asian	More Asian	LessPopinflow	MorePopinflow
QOF 2006/7 total points	0.00253***	0.00139***	0.00213***	0.00240***	0.00218***	0.00248***
	(0.00018)	(0.00029)	(0.00020)	(0.00020)	(0.00019)	(0.00025)
Distance	−0.7360***	−0.8782***	−0.7787***	−0.7607***	−0.7789***	−0.6482***
	(0.0178)	(0.0340)	(0.0179)	(0.0319)	(0.0178)	(0.0367)
Practice in different PCT	−0.863***	−0.963***	−0.967***	−0.609***	−0.896***	−0.795***
	(0.04759)	(0.12289)	(0.05478)	(0.06546)	(0.04863)	(0.09739)
Average GP age (year)	−0.0273***	−0.0212***	−0.0268***	−0.0230***	−0.0269***	−0.0221***
	(0.00187)	(0.00321)	(0.00209)	(0.00221)	(0.00195)	(0.00264)
Prop female GPs	0.259***	0.166**	0.141***	0.486***	0.237***	0.232***
	(0.03949)	(0.05964)	(0.04250)	(0.04716)	(0.04094)	(0.05700)
Prop Non‐Europ qualif GPs	−0.557***	−0.429***	−0.579***	−0.421***	−0.565***	−0.417***
	(0.03198)	(0.05832)	(0.03739)	(0.03780)	(0.03460)	(0.04175)
Opted out	0.154***	0.240**	0.154**	0.156**	0.139**	0.253***
	(0.04088)	(0.08859)	(0.05168)	(0.05022)	(0.04248)	(0.07483)
PMS contract	0.136***	0.247**	0.144**	0.152***	0.117**	0.273***
	(0.03566)	(0.08161)	(0.04852)	(0.03859)	(0.03712)	(0.06941)
MRS	0.00343***	0.00158***	0.00274***	0.00315***	0.00280***	0.00382***
	(0.00026)	(0.00034)	(0.00027)	(0.00029)	(0.00025)	(0.00045)
BIC	9,226,183	2,362,093	8,421,525	3,148,128	8,897,398	2,685,028
McFadden R^2^	0.3967	0.4109	0.4322	0.2955	0.4217	0.3142
*N* LSOA	2,296	574	2,294	576	2,295	575
*N* GP practices	966	765	970	583	973	644
*N* patients	2,696,060	654,501	2,682,712	667,849	2,705,051	645,510

Notes

Conditional logit models of practice choice for patients 24+. All models contain same covariates as model in Table 2. Distance coefficient is ${\hat{\beta }}_{d}+2{\hat{\beta }}_{d^{2}}\overset{¯}{d}+3{\hat{\beta }}_{d^{3}}\bar{d^{2}}$ where $\overset{¯}{d}$ and $\bar{d^{2}}$ are computed as the mean of the LSOA centroid to practice distance and squared distance for the full sample of 2,870 LSOAs. MRS is the coefficient on quality divided by the distance coefficient. Urban LSOAs are LSOAs classified as Urban or Town and Rural LSOAs are those classified as Village or Hamlet and Isolated Dwelling. Lower inc depriv: LSOAs in bottom 4 quintiles of income deprivation. Higher inc depriv: LSOAs in top quintile of income deprivation. Better education: LSOAs in bottom 4 quintiles of proportion of population with no formal educational qualifications. Less Educ: LSOAs in top quintile of educational deprivation. Better health: LSOAs in top four quintiles of proportion reporting being in good or fair health. Worse Health: LSOAs in bottom quintile of proportion reporting being in good or fair health. Inflow: proportion of LSOA newly resident in LSOA mid 2009 to mid 2010. Less Asian: LSOAs in bottom four quintiles of proportion of LSOA population classified as Asian or Asian British. More Asian: LSOAs in top quintile of LSOA population classified as Asian or British Asian. Less popinflow: LSOAs in bottom four quintiles of population inflow distribution. More popinflow: LSOAs in top quintile of inflow distribution. Conditional logit models of choice of practice. All models have the same covariates as in Table 2. The distance coefficient is the derivative of the utility function evaluated using the estimated coefficients and the mean of the powers of LSOA to practice distance for the whole sample. Standard errors clustered at LSOA level are in parentheses. *p < 0.05, **p < 0.01, ***p < 0.001.

**Table C11:** Choice Models with Different Choice Set Radii and Stratified By LSOA Population Growth: Full Results

	Choice set radius						Population growth	
	10 km	8 km	6 km	4 km	2 km	Urban 3 km Rural 7 km	Lower 4 quintiles	Top quintile
QOF 2006/7 total points	0.00222***	0.00217***	0.00217***	0.00230***	0.00215***	0.00219***	0.00214***	0.00286***
	(0.00016)	(0.00015)	(0.00015)	(0.00016)	(0.00018)	(0.00016)	−0.00016	−0.00042
Distance km	−1.606***	−1.549***	−1.461***	−1.439***	−1.478***	−1.652***	−1.621***	−1.529***
	(0.04044)	(0.04881)	(0.06782)	(0.10411)	(0.27757)	(0.06498)	(0.04467)	(0.09251)
Distance squared km	0.126***	0.103***	0.0588*	0.0352	−0.0771	0.152***	0.127***	0.117***
	(0.01189)	(0.01661)	(0.02879)	(0.05955)	(0.28342)	(0.02721)	(0.01343)	(0.02478)
Distance cubic km	−0.00447***	−0.00229	0.00325	0.00767	0.0708	−0.00616*	−0.0044***	−0.00445*
	(0.00091)	(0.00154)	(0.00346)	(0.00993)	(0.08697)	(0.00290)	(0.0011)	(0.00178)
Practice in different PCT	−0.881***	−0.862***	−0.862***	−0.817***	−0.655***	−0.818***	−0.901***	−0.796***
	(0.04436)	(0.04551)	(0.04574)	(0.04751)	(0.06690)	(0.05238)	(0.04757)	(0.10922)
Average GP age	−0.0257***	−0.0258***	−0.0252***	−0.0245***	−0.0245***	−0.0254***	−0.0230***	−0.0347***
	(0.00163)	(0.00164)	(0.00165)	(0.00169)	(0.00188)	(0.00173)	(0.00174)	(0.00431)
Prop female GPs	0.239***	0.243***	0.260***	0.278***	0.351***	0.294***	0.303***	0.0293
	(0.03364)	(0.03388)	(0.03366)	(0.03436)	(0.04089)	(0.03584)	(0.03529)	(0.08602)
Prop Non‐Eur qualif GPs	−0.527***	−0.522***	−0.521***	−0.497***	−0.396***	−0.498***	−0.265***	−0.340***
	(0.02809)	(0.02842)	(0.02853)	(0.02913)	(0.03178)	(0.03013)	(0.01692)	(0.03255)
Opted out	0.160***	0.173***	0.196***	0.183***	0.184***	0.174***	0.163***	0.189*
	(0.03748)	(0.03762)	(0.03752)	(0.03823)	(0.04495)	(0.03945)	(0.04166)	(0.08421)
PMS contract	0.148***	0.168***	0.184***	0.174***	0.148***	0.157***	0.122***	0.248***
	(0.03317)	(0.03307)	(0.03320)	(0.03364)	(0.03932)	(0.03450)	(0.03713)	(0.07025)
Distance	−0.7512***	−0.7717***	−0.7457***	−0.6715***	−2.0356	−0.6631***	−0.7506***	−0.7554***
	(0.0163)	(0.0167)	(0.0273)	(0.1728)	(2.9421)	(0.0255)	(0.0184	(0.0337)
MRS	0.00296***	0.00281***	0.00291***	0.00342***	−0.00106	0.00331***	−0.00277***	−0.00377***
	(0.00022)	(0.00021)	(0.00023)	(0.00091)	(0.00153)	(0.00027)	(0.00023	(0.00058)
McFadden R^2^	0.3991	0.3665	0.3151	0.2345	0.1081	0.2355	0.3936	0.4225
*N* LSOA	2,870	2,806	2,670	2,428	1,925	2,676	2,296	574
*N* practices	973	923	853	738	605	836	945	772
*N* patients	3,291,581	3,190,284	3,003,036	2,661,832	1,844,207	2,773,892	2,680,561	670,000

Notes

Conditional logit models of choice of practice. Choice sets are all practices within stated distance of LSOA centroid and, if there are more than 30 such, the 30 nearest to the LSOA centroid. Choice set for the models stratified by LSOA population growth is all practice within 10 km, and if there are more than 30 such, the 30 nearest to the LSOA centroid. All models have the same covariates and cubic distance specification as Table 2. The distance coefficient is ${\hat{\beta }}_{d}+2{\hat{\beta }}_{d^{2}}\overset{¯}{d}+3{\hat{\beta }}_{d^{3}}\bar{d^{2}}$ where $\overset{¯}{d}$ and $\bar{d^{2}}$ are computed as the mean of the LSOA centroid to practice distance and squared distance for the sample defined by the stated radius. MRS is the quality coefficient divided by the distance coefficient. Conditional logit models of choice of practice. All models have the same covariates as in Table 2. The distance coefficient is the derivative of the utility function evaluated using the estimated coefficients and the mean of the powers of LSOA to practice distance for the whole sample. Standard errors clustered at LSOA level are in parentheses. *p < 0.05, **p < 0.01, ***p < 0.001.

**Table C12:** Choice Model with Instrumented General Practice Quality: Full Results

	IV: mean quality all other practices in PCT	IV: mean quality of non‐rivals in PCT
	First‐stage model: practice quality (OLS)	First‐stage model: practice quality (OLS)
	Coefficient	Coefficient
IV	0.530***	0.323
	(128.8)	(0.216)
Different PCT	9.672	1.452
	(6.048)	(11.152)
Distance	−36.23	−42.18
	(919.393)	(30.02)
Distance squared	5.668	6.307
	(3.57)	(5.953)
Distance cubed	−0.253	−0.241
	(0.205)	(0.357)
Av GP age months	−0.1045**	−0.1112*
	(0.0350)	(0.0438)
Percentage of female GPs	0.1295	0.2151
	(0.0950)	(0.1393)
Percentage of non‐European qualified GPs	0.1927**	−0.2208
	(0.07600)	(0.1131)
Opted out	11.34*	7.406
	(4.75)	(7.6220)
PMS contract	10.08	11.41
	(5.25)	(7.65)
Constant	552.4***	759.3**
	(128.8)	(218.2)
F test on IV	16.53	2.22

	Second stage: choice of practice (conditional logit)		Second stage: choice of practice (conditional logit)	
	Full sample model coefficient	Mean coef bootstrap samples (SD of coef in bootstrap samples)	Full sample model coefficient	Mean coef bootstrap samples (SD of coef in bootstrap samples)
QOF 2006/7 total points	0.00622**	0.00615	0.0108***	0.01080
	(0.00211)	(0.00215)	(0.0025)	(0.00264)
1st stage residual QOF 2006/7	−0.00398	−0.00390	−0.00855***	−0.00855
	(0.00209)	(0.00212)	(0.00253)	(0.00263)
Distance	−0.7517***	−0.75071	−0.7803***	−0.77919
	(0.0163)	(0.01602)	(0.0177)	(0.01786)
Practice in different PCT	−0.882***	−0.87626	−0.921***	−0.91372
	(0.044)	(0.03927)	(0.056)	(0.04530)
Average GP Age (months)	−0.00175***	−0.00172	−0.00185***	−0.00184
	(0.00025)	(0.00027)	(0.00033)	(0.00034)
Percentage of female GPs	0.00194***	0.00199	−0.000358	−0.00031
	(0.00041)	(0.00035)	(0.000639)	(0.00065)
Percentage of non‐European qualified GPs	−0.00448***	−0.00450	−0.00289***	−0.00286
	(0.00051)	(0.00053)	(0.00065)	(0.00069)
Opted out	0.115**	0.10720	−0.00397	−0.01332
	(0.043)	(0.04163)	(0.04991)	(0.04564)
PMS contract	0.106**	0.09971	−0.0283	−0.03597
	(0.038)	(0.03821)	(0.0524)	(0.04823)
BIC	11596983	38756048	8126726729	31786655
McFadden R^2^	0.3992	0.39903	0.4224	0.42252
MRS	0.0083**	0.00821	0.0138***	0.01387***
	(0.0028)	(0.00288)	(0.0032)	(0.00342)

Notes

First‐stage model: practice QOF 2006/7 points regressed on mean of QOF 2006/7 points of all other practices in the same PCT or on the mean of QOF 2006/7 points for non‐rival practices in the same PCT and on means of all other explanatories in practice choice model taken over all LSOAs whose choice set includes the practice, with SEs clustered on PCTs. Second‐stage models: conditional logit models of practice choice with all explanatories from model in Table 2 plus quality residuals from first stage model. For the 2SRI model with the average quality of non‐rivals as the IV we drop 333 practices whose PCTs contain only rivals and we drop 122 LSOAs whose choice sets contain only the dropped practices. For the second‐stage practice choice models, we report the coefficients and SEs from the 2SRI model estimated with the full sample of LSOAs, the average of the coefficients estimated in the bootstrap samples, and the standard deviation of the coefficient from bootstrap model. The coefficient on distance is ${\hat{\beta }}_{d}+2{\hat{\beta }}_{d^{2}}\overset{¯}{d}+3{\hat{\beta }}_{d^{3}}\bar{d^{2}}$ where $\overset{¯}{d}$ and $\bar{d^{2}}$ are computed as the mean of the LSOA centroid to practice distance and squared distance for the whole sample. MRS is the coefficient on distance divided by the coefficient on quality. Conditional logit models of choice of practice. All models have the same covariates as in Table 2. The distance coefficient is the derivative of the utility function evaluated using the estimated coefficients and the mean of the powers of LSOA to practice distance for the whole sample. Standard errors clustered at LSOA level are in parentheses. *p < 0.05, **p < 0.01, ***p < 0.001.
