# Supplementary material for: Does Quality Affect Patients’ Choice of Doctor? Evidence from England
Source: Econ J (London). 2016 Feb 23;127(600):445–94. doi: 10.1111/ecoj.12282 (PMC5349292; doi:10.1111/ecoj.12282)
Supplement: Supplementary file 2 [file ECOJ-127-445-s002.pdf]

Does quality affect patients' choice of doctor? Evidence from England

Replication instructions

Data sets:

The Attribution Data Set is from NHS England. The organisation does not allow the transfer of the data set, potential users must apply near a NHS England Information Governance Senior Manager.

Lower Super Output Areas geographical data is available at https://geoportal.statistics.gov.uk/

Location of practices – Data sources describe in the paper and we have included a do file on how to build it (**GP_network_for_EJ_22Feb2015.do**)

General Medical Service data must be obtained by data sharing agreement between the potential user and the Data Access Request Service team of HSCIC.

GP Patient survey data is available at https://gp-patient.co.uk/about

Number of Ambulatory Care Sensitive Conditions admission per GP practice is built from Hospital Episodes Statistics. HSCIC doesn't allow us to share the data, so potential user need to apply for the data near HSCIC. We do provide the do file (ACSCs200607_as_qual_GPprac.do) we used to construct the Number of Ambulatory Care Sensitive Conditions admission per GP practice from HES raw data.

Building the data set:

Building_the_dataset_forEJ.do

The do file merges the different data sets and builds the choice set for each LSOA.

Papers results:

The tables in the paper can be replicated using the do file: **tables_for_EJ_paper.do**

We have flagged in red when a separate do file has been used.

* table 1 : descriptive statistics

* table 2 : preferred model - cubic distance & qof 2006 total points

* table 3 : comparison of practice choice models by age and gender groups

* table 4 : choice models for LSOA samples stratified by socio-economic characteristics and population mobility

* table 5: choice model: mixed and conditional logit specification

* table 6: choice models with different choice set radii and stratified by LSOA population growth

* table 7 : choice model with instrumented general practice quality – only second stage (first stage in another do file - **table7_and_table12_1ststage_plus_A4.do**)

* table 8 : effect sizes for quality and other practice characteristics

Figures

The do files used to produce each figure are noted in red.

* Figure 1: Map done in ArcGIS – geographical data (shape files) available on request to authors.

* Figure 2: Distributions of distance to chosen practice – separate do file **figure2_distance_to_choosen_practice.do**

*Figure 3: Estimates of the average marginal effects of distance – separate do file **figure3_estimates of AME effects of distance.do**

* Figure 4: Plot of cross practice quality elasticities against distance to other practice – separate do file **Figure4_Cross_elasticities.do**

* Figure 5: Effect of quality information on predicted number of patients in practices – separate do file **Figure5_effects_of_quality_information_on_predicted_number_of_patientes_in_practices.do**

*** (a) Distribution of percentage change in predicted number of patients (b)

*** (b) Log percentage change in predicted patient numbers and 2006/7 QOF points

Appendix:

The tables in appendix can be replicated using the do file **tables_for_Appendix_EJ_paper.do.** We have flagged in red when a separate do file has been used.

* table A3: Descriptive Stats for quality measures - code included in the table 1 code (**tables_for_EJ_paper.do**)

* table A4: correlation between quality measures – in separate do file (table7_and_table12_1ststage_plus_A4.do)

* table A5 : choice models with alternative QOF points based quality measures

* table A6: Choice models with other QOF derived quality measures and with ACSC emergency admissions

* table A7: Choice models patient reported experience quality measures

* table A8: Choice models with alternative distance specifications

* table A9 : Comparison of practice choice models by age and gender group: full results - - code included in **tables_for_EJ_paper.do**

* table A10: Choice models for LSOA samples stratified by socio-economic characteristics and population mobility: full results - - code included in **tables_for_EJ_paper.do**

* table A11 : choice models with different choice set radii and stratified by LSOA population growth: full results - - code included in **tables_for_EJ_paper.do**

* table A12 : choice model with instrumented general practice quality: full results – separate do file (**table7_and_table12_1ststage_plus_A4.do**)
